# Associations of face-to-face and non-face-to-face social isolation with all-cause and cause-specific mortality: 13-year follow-up of the Guangzhou Biobank Cohort study

**DOI:** 10.1186/s12916-022-02368-3

**Published:** 2022-05-02

**Authors:** Jiao Wang, Wei Sen Zhang, Chao Qiang Jiang, Feng Zhu, Ya Li Jin, Kar Keung Cheng, Tai Hing Lam, Lin Xu

**Affiliations:** 1 School of Public Health, Sun Yat-sen University (North Campus), No. 74, 2nd Zhongshan Road, Guangzhou, Guangdong China; 2 Guangzhou Twelfth People’s Hospital, Guangzhou, China; 3 Institute of Applied Health Research, University of Birmingham, Birmingham, UK; 4 School of Public Health, The University of Hong Kong, Hong Kong, China

**Keywords:** Social isolation, Face-to-face contact, Non-face-to-face contact, Mortality, Cardiovascular disease

## Abstract

**Background:**

Although social isolation has been associated with a higher mortality risk, little is known about the potential different impacts of face-to-face and non-face-to-face isolation on mortality. We examined the prospective associations of four types of social isolation, including face-to-face isolation with co-inhabitants and non-co-inhabitants, non-face-to-face isolation, and club/organization isolation, with all-cause and cause-specific mortality separately.

**Methods:**

This prospective cohort study included 30,430 adults in Guangzhou Biobank Cohort Study (GBCS), who were recruited during 2003–2008 and followed up till Dec 2019.

**Results:**

During an average of 13.2 years of follow-up, 4933 deaths occurred during 396,466 person-years. Participants who lived alone had higher risks of all-cause (adjusted hazard ratio (AHR) 1.24; 95% confidence interval (CI) 1.04-1.49) and cardiovascular disease (CVD) (1.61; 1.20–2.03) mortality than those who had ≥ 3 co-habitant contact after adjustment for thirteen potential confounders. Compared with those who had ≥ 1 time/month non-co-inhabitant contact, those without such contact had higher risks of all-cause (1.60; 1.20–2.00) and CVD (1.91; 1.20–2.62) mortality. The corresponding AHR (95% CI) in participants without telephone/mail contact were 1.27 (1.14–1.42) for all-cause, 1.30 (1.08–1.56) for CVD, and 1.37 (1.12–1.67) for other-cause mortality. However, no association of club/organization contact with the above mortality and no association of all four types of isolation with cancer mortality were found.

**Conclusions:**

In this cohort study, face-to-face and non-face-to-face isolation were both positively associated with all-cause, CVD-, and other-cause (but not cancer) mortality. Our finding suggests a need to promote non-face-to-face contact among middle-aged and older adults.

**Supplementary Information:**

The online version contains supplementary material available at 10.1186/s12916-022-02368-3.

## Background

Social isolation is defined as “a state in which the individual lacks a sense of belonging socially, lacks engagement with others, has a minimal number of social contact and they are deficient in fulfilling and quality relationships” [1, 2]. It has been shown to be associated with higher risks of heart disease and stroke [3], dementia [4], and mortality [5]. Though the adverse effect on mortality was comparable to or greater than some well-established risk factors [6, 7], it has received less attention [8]. Social isolation is a growing epidemic in older people. For example, the prevalence is 24% in the USA [9], 10–43% in North America [2], 20% in India [10], and 33.1% in China [11]. During the coronavirus disease (COVID)-19 pandemic, “social distancing” measures have aggravated pre-existing social isolation [12]. The American Association of Retired Persons reported that 14% of adults aged 50+ years in the USA were socially isolated in 2017, and the percentage reached 61% in 2020 since the pandemic began [12], when face-to-face contact is heavily restricted, and non-face-to-face contact (i.e., conventionally by telephone and letter, or recently by e-mail and social media) become the predominant forms of social interactions. However, the associations of face-to-face and non-face-to-face contact with mortality have not been well-defined and differentiated in previous studies.

A meta-analysis of 70 prospective studies published up to 2014 reported 29% higher risk of mortality associated with social isolation and loneliness [13], and another meta-analysis of 91 studies up to 2015 reported 13% higher risk of mortality associated with lower levels of social contact frequency [14]. But no study in the two meta-analysis papers above reported face-to-face and non-face-to-face contact separately. We found thirty studies published after these two meta-analyses (Additional file 1: Table S1) [5, 15–43]. Of them, only one study (*n* = 1023) classified participants according to social isolation types (face-to-face and non-face-to-face) [30]. However, this study only reported the all-cause (no CVD and cancer) mortality risk related to the co-existence of social (non-face-to-face) isolation and homebound status but did not report results of non-face-to-face contact separately [30]. In the context of physiological mechanisms, a previous study showed that only in-person contacts, but not virtual contact, protected against mood disorders [44], which indicated non-face-to-face contacts might have a different effect on human health, even though face-to-face contacts have been repeatedly linked to better health outcomes [5, 32].

We investigated the prospective associations of face-to-face and non-face-to-face social isolation with all-cause and cause-specific mortality in 30,430 participants who were recruited in 2003–2008 and had all-cause and cause-specific mortality follow-up data up to December 2019 in the Guangzhou Biobank Cohort Study (GBCS).

## Methods

### Study participants

The Guangzhou Biobank Cohort Study (GBCS) in China is a 3-way collaborative project among the Guangzhou Twelfth People’s Hospital and the Universities of Hong Kong and Birmingham. Participants were recruited from a community social and welfare association, the “Guangzhou Health and Happiness Association for the Respectable Elders” (GHHARE). From 2003 to 2008, 32,850 members of the GHHARE were invited, and of them, 30,430 agreed to participate and signed informed consents, with a response rate of 92.6%. GHHARE is a large unofficial organization with ten branches throughout all districts of Guangzhou. Membership of this association is open to Guangzhou residents aged 50 years or older for a nominal, monthly fee of 4 Chinese yuan renminbi (CNY) (about 50 US cents). Seven percent of local residents aged 50+ years enrolled in the GHHARE. All surviving participants were invited to return for follow-up examination from March 2008 to December 2012. Details of the GBCS, GHHARE, and some prospective study results have been reported previously [45–47]. Briefly, baseline information was collected using a computer-based questionnaire in face-to-face interviews by trained nurses in the Guangzhou Twelfth People’s Hospital. Information of demographic characteristics, lifestyle factors, family and personal medical history, and anthropometrics, blood pressure, fasting plasma glucose, lipids, and inflammatory markers was collected following standard protocols. The reliability and validity of the questionnaire were tested 6 months into recruitment by recalling 200 randomly selected participants for re-interview, and the results were satisfactory [45].

### Social isolation measurement

**Fig. 1:**
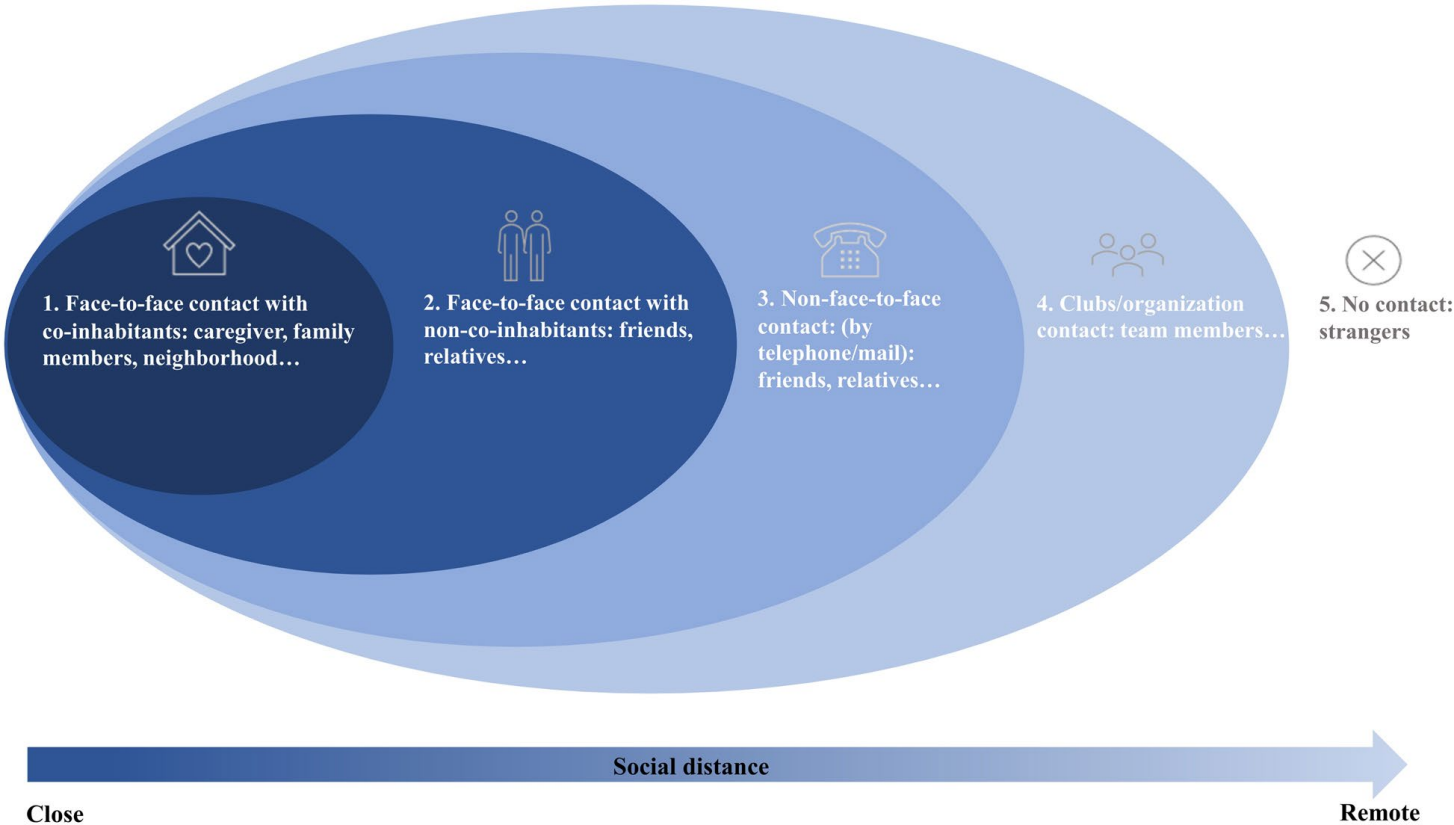
The four types of social contact

Since no study has reported face-to-face and non-face-to-face contact separately, we first proposed the 4 types of social contact using the validated questions in the Berkman-Syme Social Network Index (SNI) [48] with appropriate revise. The four types of social contact were illustrated in Fig. 1 and described in Table 1. We included mail as another way of non-face-to-face contact besides telephone, since the telephone and mail are the commonest non-face-to-face ways to contact others in 2003, before smartphone, Internet, and social media had become popular. A composite social isolation score was derived from the sum of four types of social isolation, with a score from 0 to 7. A higher score indicates greater social isolation (Table 1).

**Table 1:** The four types of social isolation question and the scoring criteria for the composite social isolation score

Items	Questions	Answers	Scoring		
			Score of 0 if:	Score of 1 if:	Score of 2 if
Face-to-face contact with co-inhabitants	How many people do you spend time with on a regular basis (at least once a month; do not include phone/mail conversations; excluding the workplace; including those who lived together)?	“≥ 3 people”, “< 3 people,” and “Live alone”	≥ 3 people	< 3 people	Live alone
Face-to-face contact with non-co-inhabitants	How often do you see any of your friends or relatives (do not include phone/mail conversations; excluding those who lived together)?	“≥1 time/month”, “< 1 time/month,” and “No such contact”	≥ 1 time /month	< 1 time/month	No such contact
Non-face-to-face contact (by telephone/mail)	Do you have any relatives or friends you don't often see but keep in contact with by phone or letter?	“≥1 time/month,” “< 1 time/month,” and “No such contact”	≥ 1 time /month	< 1 time/month	No such contact
Club/organization contact	How often do you join in activities of the GHHARE or other organizations?	“≥1 time/month” and “< 1 time/month”	≥ 1 time /month	< 1 time/month	NA
Composite score: sum of scores			0~7		

### Mortality

As described in our previous papers [49, 50], information on causes of death up to December 31, 2019, was obtained via record linkage with the Death Registry of the Center for Disease Control and Prevention in Guangzhou. Causes of death were coded according to the 10th Revisions of the International Classification of Diseases (ICD-10) by trained clinical coding officers in each hospital. The ICD-10 codes of the cause-specific mortality were as follows: cardiovascular disease (I00-I99, excluding I26, I27), cancer (C00-C96), and other diseases (all remaining ICD-10 codes). A physician panel including 5 chief physicians from various disciplines reviewed all available medical records of the same individuals and assigned in a standard manner a cause of death, with the assistance of an epidemiologist in the last meeting for unsettled cases.

### Potential confounders

To examine the extent to which baseline socioeconomic position (SEP), biological, behavioral, and psychological factors explained the associations, we included them in two models. Sex, age, and self-rated health (good/very good, poor/very poor) were included in minimal model. The fully adjusted model was adjusted for thirteen factors (sex, age, self-rated health, SEP, biological and behavioral factors): (1) SEP: education (primary and below, middle school, and college or above), occupation (manual, non-manual, and others), family income (≤ 30,000 CNY/year, > 30,000 CNY/year, and not known; US$1 = 7 CNY); (2) biological factors: height and weight were measured, and body mass index (BMI) was calculated by weight in kilogram divided by height in meters squared (kg/m^2^). After an initial 5-min rest, seated blood pressure was measured 3 times at 1–5 min’s intervals using an Omron 705CP sphygmomanometer (Omron Corp, Kyoto, Japan). Systolic and diastolic blood pressure (SBP and DBP) were calculated as the average of the last 2 measurements. Fasting glucose was measured after an overnight fast; (3) behavioral factors: smoking status (never, former and current), alcohol use (never, former, and current drinkers), physical activity assessed by International Physical Activity Questionnaire (inactive, moderate, and active) [46]; (4) psychological factors: stress level assessed by Perceived Stress Scale-14 items (PSS-14) [51], and cognitive function assessed by Delayed Word Recall Test (DWRT) [52]. The DWRT was assessed in the full dataset, but PSS-14 was only assessed in a subsample of GBCS in phrases 1 and 2 (*n* = 19,947). All potential confounders were categorized as in Table 2. We calculated the percentage of excess risk mediated (PERM), which represented the percentage of risks that explained by SEP, and biological and behavioral factors as in the two previous UK Biobank papers [5, 32]:

$$\mathrm{PERM}=\frac{\left[\mathrm{Hazard}\ \mathrm{ratio}\ \left(\mathrm{minimally}\ \mathrm{adjusted}\right)-\mathrm{hazard}\ \mathrm{ratio}\ \left(\mathrm{fully}\ \mathrm{adjusted}\ \mathrm{adjusted}\right)\right]}{\mathrm{Hazard}\ \mathrm{ratio}\left(\mathrm{minimally}\ \mathrm{adjusted}\right)-1}\ast 100\%$$

**Table 2:** Baseline characteristics of the participants by four types of social contact isolation in Guangzhou Biobank Cohort study

	Face-to-face contact with co-inhabitants			Face-to-face contact with non-co-inhabitants			Non-face-to-face contact (by telephone/mail)			Club/organization contact	
	≥ 3 people	< 3 people	Live alone	≥ 1 time/month	< 1time/month	No such contact	≥ 1 time/month	< 1 time/month	No such contact	≥ 1 time/month	< 1 time/month
Number of participants	27,701	1926	647	26,438	3582	160	23,557	4913	1707	13,873	16,253
Sex, men %	7332 (26.9)	644 (33.4)	198 (30.6) **	7115 (26.9)	1164 (32.5)	58 (36.3) **	6199 (26.3)	1606 (32.7)	530 (31.1) **	3449 (24.9)	4879 (30.0) **
Age, years, mean (SD)	61.9 (7.0)	62.9 (7.7)	64.2 (8.3) **	61.9 (7.1)	62.9 (7.0)	64.3 (7.9) **	61.8 (7.1)	62.7 (7.1)	63.8 (7.7) **	62.9 (7.1)	61.3 (7.1) **
Self-rated health, good/very good, %	22,183 (82.9)	1397 (75.7)	418 (69.7) **	21,498 (83.0)	2676 (76.8)	108 (69.7) **	19,270 (83.5)	3737 (77.8)	1269 (76.7) **	11,401 (83.7)	12,836 (80.9) **
**Socioeconomic position**											
Education, %											
≤ Primary	11,456 (42.1)	923 (48.0)	389 (60.1) **	11,158 (42.2)	1707 (47.7)	89 (56.0) **	9478 (40.3)	2387 (48.6)	1087 (63.7) **	5766 (41.6)	7173 (44.2) **
Middle school	13,262 (48.7)	884 (45.9)	224 (34.6)	12,902 (48.8)	1555 (43.4)	61 (38.4)	11,833 (50.3)	2142 (43.6)	543 (31.8)	6748 (48.7)	7738 (48.1)
≥ College	2506 (9.2)	119 (6.2)	34 (5.3)	2370 (9.0)	318 (8.8)	9 (5.7)	2239 (9.5)	381 (7.8)	76 (4.5)	1354 (9.8)	1336 (8.2)
Occupation, %											
Manual	16,423 (60.6)	1256 (65.7)	463 (72.5) **	15,947 (60.65)	2295 (64.3)	110 (69.2) **	13,952 (59.6)	3118 (63.7)	1284 (75.7) **	8233 (59.7)	10,095 (62.4) **
Non-manual	6532 (24.1)	415 (21.7)	93 (14.6)	6329 (24.1)	794 (22.2)	33 (20.8)	5883 (25.1)	1049 (21.4)	222 (13.1)	3848 (27.9)	3288 (20.3)
Others	4146 (15.3)	240 (12.6)	83 (12.9)	4017 (15.3)	481 (13.5)	16 (10.1)	3593 (15.3)	728 (14.9)	191 (11.3)	1720 (12.5)	2786 (17.2)
Family income, CNY/year, %											
≤ 30,000	10,191 (37.5)	826 (43.1)	271 (42.0) **	9978 (37.8)	1409 (39.4)	78 (48.8) **	8793 (37.4)	1912 (39.0)	761 (44.7) **	5351 (38.6)	6099 (37,6) **
> 30,000	10,534 (38.7)	659 (34.2)	186 (28.8)	10,207 (38.7)	1250 (34.9)	38 (23.8)	9300 (39.5)	1715 (34.9)	478 (28.1)	5055 (36.5)	6415 (39.5)
Do not know	6478 (23.9)	436 (22.8)	190 (29.2)	62,20 (23.6)	919 (25.7)	44 (27.5)	5436 (23.1)	1281 (26.1)	464 (27.3)	3451 (24.9)	3718 (22.9)
**Biological factors**											
BMI, kg/m^2^, mean (SD)	23.8 (3.3)	23.7 (3.3)	23.7 (3.2)	23.8 (3.3)	23.8 (3.4)	23.2 (3.3)	23.8 (3.3)	23.7 (3.3)	23.6 (3.4) *	23.8 (3.3)	23.7 (3.3)
SBP, mmHg, mean (SD)	130.2 (22.0)	131.3 (22.8)	133.3 (23.0) *	130.3 (22.0)	131.1 (23.0)	130.1 (22.7)	130.0 (21.9)	131.2 (22.7)	133.7 (23.4) *	131.7 (22.2)	129.3 (22.0)
DBP, mmHg, mean (SD)	73.6 (11.2)	73.8 (11.4)	73.6 (11.3)	73.6 (11.2)	73.8 (11.6)	72.9 (11.0)	73.6 (11.2)	73.8 (11.4)	74.0 (11.5)	73.9 (11.2)	73.4 (11.3)
FG, mmol/L, mean (SD)	5.7 (1.6)	5.8 (1.7)	5.9 (1.7) *	5.7 (1.7)	5.8 (1.7)	6.0 (2.4)	5.7 (1.6)	5.8 (1.7)	5.8 (1.9)	5.8 (1.6)	5.7 (1.7)
**Behavioral factors**											
Smoking status, %											
Never	21,171 (81.5)	1464 (76.2)	486 (75.4) **	21,497 (81.4)	2757 (77.2)	109 (68.1) **	19,280 (81.9)	3766 (76.8)	1317 (77.3) **	11,544 (83.3)	12,776 (78.7) **
Former	2396 (8.8)	230 (11.8)	78 (12.1)	2350 (8.9)	393 (11.0)	21 (13.1)	2076 (8.8)	526 (10.7)	160 (9.4)	1243 (9.0)	1517 (9.3)
Current	2643 (9.7)	229 (11.9)	81 (12.6)	2569 (9.7)	423 (11.8)	30 (18.8)	2180 (9.3)	615 (12.5)	226 (13.3)	1070 (7.7)	1945 (12.0)
Alcohol use, %											
Never	19,926 (73.5)	1205 (62.9)	315 (50.2) **	19,152 (72.8)	2472 (69.4)	84 (52.5) **	17,093 (72.9)	3397 (69.6)	1216 (71.5) **	10,114 (73.3)	11,560 (71.5) **
Former	902 (3.3)	89 (4.7)	47 (7.5)	935 (3.55)	113 (3.17)	13 (8.1)	769 (3.3)	224 (4.6)	69 (4.1)	404 (2.9)	658 (4.1)
Current	6285 (23.2)	621 (32.4)	267 (42.3)	6220 (23.6)	975 (27.4)	63 (39.4)	5581 (23.8)	1259 (25.8)	417 (24.5)	3280 (23.8)	3957 (24.5)
Physical activity, %											
Inactive	2246 (8.3)	142 (7.3)	25 (3.8) **	2091 (7.9)	335 (9.4)	5 (3.1) **	1862 (7.9)	450 (9.2)	117 (6.9) **	653 (4.7)	1778 (10.9) **
Minimally active	11,382 (41.8)	657 (34.1)	132 (20.4)	10,880 (41.2)	1385 (38.7)	63 (39.4)	9439 (40.1)	2099 (42.7)	790 (46.3)	5006 (36.1)	7308 (45.0)
Active	13,607 (50.0)	1127 (58.5)	490 (75.7)	13,467 (50.9)	1862 (52.0)	92 (57.5)	12,256 (52.0)	2364 (48.1)	800 (46.9)	8214 (59.2)	7167 (44.1)
**Psychological factors**											
Stress level (PPS-14), mean (SD) (*n* = 19,947)	14.7 (4.3)	15.8 (4.5)	16.8 (5.0) **	14.7 (4.3)	14.8 (4.6)	15.6 (5.4) **	14.7 (4.2)	14.9 (4.6)	15.4 (5.1) **	14.9 (4.2)	14.6 (4.4) **
Cognitive function (DWRT), mean (SD)	5.5 (1.8)	5.4 (2.0)	5.3 (2.2) **	5.5 (1.8)	5.3 (1.9)	4.6 (2.3) **	5.6 (1.8)	5.4 (1.8)	5.0 (2.0) **	5.6 (1.8)	5.4 (1.9) **

*P < 0.05; **P < 0.001

*CNY* Chinese Yuan Renminbi (US$1 = 7 CNY), *PSS-14* Perceived Stress Scale-14 items, *DWRT* Delayed Word Recall Test, *SD* standard deviation, *BMI* body mass index, *SBP* systolic blood pressure, *DBP* diastolic blood pressure, *FG* fasting glucose

### Statistical analysis

Chi-square test or analysis of variance was used to compare baseline characteristics by four types of social isolation. Associations of four types of social isolation with all-cause and cause-specific mortality were estimated by Cox proportional hazards model giving hazard ratios (HRs) and 95% confidence intervals (CI). Schoenfeld’s residuals were used to assess the proportional hazard assumption, and no major violations were observed. In sensitivity analysis, competing risk analysis (Fine-Gray’s model) [53] were used to assess the association of social isolation with cause-specific mortality, where each cause was simultaneously modeled as a different event. In sensitivity analysis, fully adjusted model was additionally adjusted for psychological factors using the subgroup dataset because PSS-14 data were available in the subsample only. To explore potential bias due to missing data, we assessed the proportion of missing data for all variables. The proportion of missing data in all variables was very low (i.e., less than 3%) (Additional file 1: Table S2). Therefore, we used complete case analysis in the current study. Subgroup analyses were done by sex, age groups (< 60 years, ≥ 60 years), education (≤ primary, ≥ middle school), and self-rated health (poor/very poor, good/very good) to investigate whether the associations of social isolation with mortality varied by these factors [32, 54, 55]. For sensitivity analysis, in order to assess the independent associations of face-to-face and non-face-to-face isolation with mortality, we analyzed the associations of 3 types of contact (excluding club/organization isolation which showed no association with mortality) with all-cause mortality after mutual adjustment in a sensitivity analysis. To partly address reverse causation, we excluded participants who died within the first 2 years in the main analysis and provided the original data (without excluding death within first 2 years) analysis for comparability. Also, a subsample of 18,104 participants who returned for repeated examination during 2008–2012 was analyzed to examine the associations in survivors after baseline examination. All statistical analyses were done using the Stata version 15.0 (STATA Corp LP). All tests were two-sided, with *P* < 0.05 as statistically significant.

## Results

**Fig. 2:**
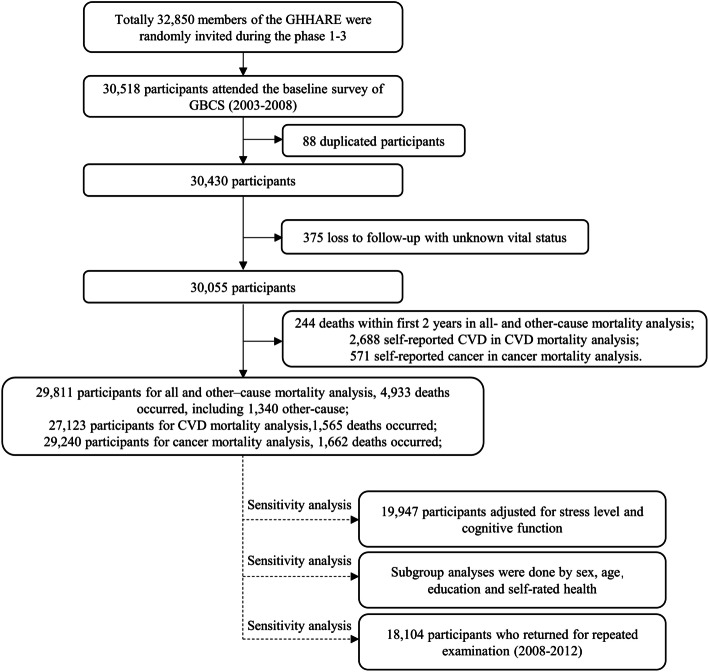
Flow diagram of the study participants

During an average follow-up of 13.2 (standard deviation = 2.8) years with 396,466 person-years, of 30,430 participants, 375 who were lost to follow-up with unknown vital status were excluded. We also excluded 244 deaths that occurred within the first 2 years. We then additionally excluded 2688 with self-reported CVD and 571 with self-reported cancer in the analyses of CVD and cancer mortality respectively. Of 29,811 participants included in the all- and other-cause analysis, up to December 2019, 4933 deaths occurred, in which 1340 were other-cause. Of 27,123 participants for CVD mortality analysis, 1565 deaths occurred, and of 29,240 participants for cancer mortality analysis, 1662 deaths occurred (Fig. 2).

Table 2 shows that those who were socially isolated (i.e., with less contact in all groups) were older and fewer had good/very good health status. More men were isolated with non-co-inhabitants and club/organization. Social isolation from face-to-face contact with co-inhabitants and non-co-inhabitants was associated with lower SEP (lower education, manual occupation, and lower family income), more smoking and alcohol use, and higher physical activity. Participants with less non-face-to-face contact and club/organization contact showed similar patterns but had lower physical activity. Moreover, those with less social contact had higher PSS-14 (stress level) and lower DWRT (cognitive function) scores.

**Fig. 3:**
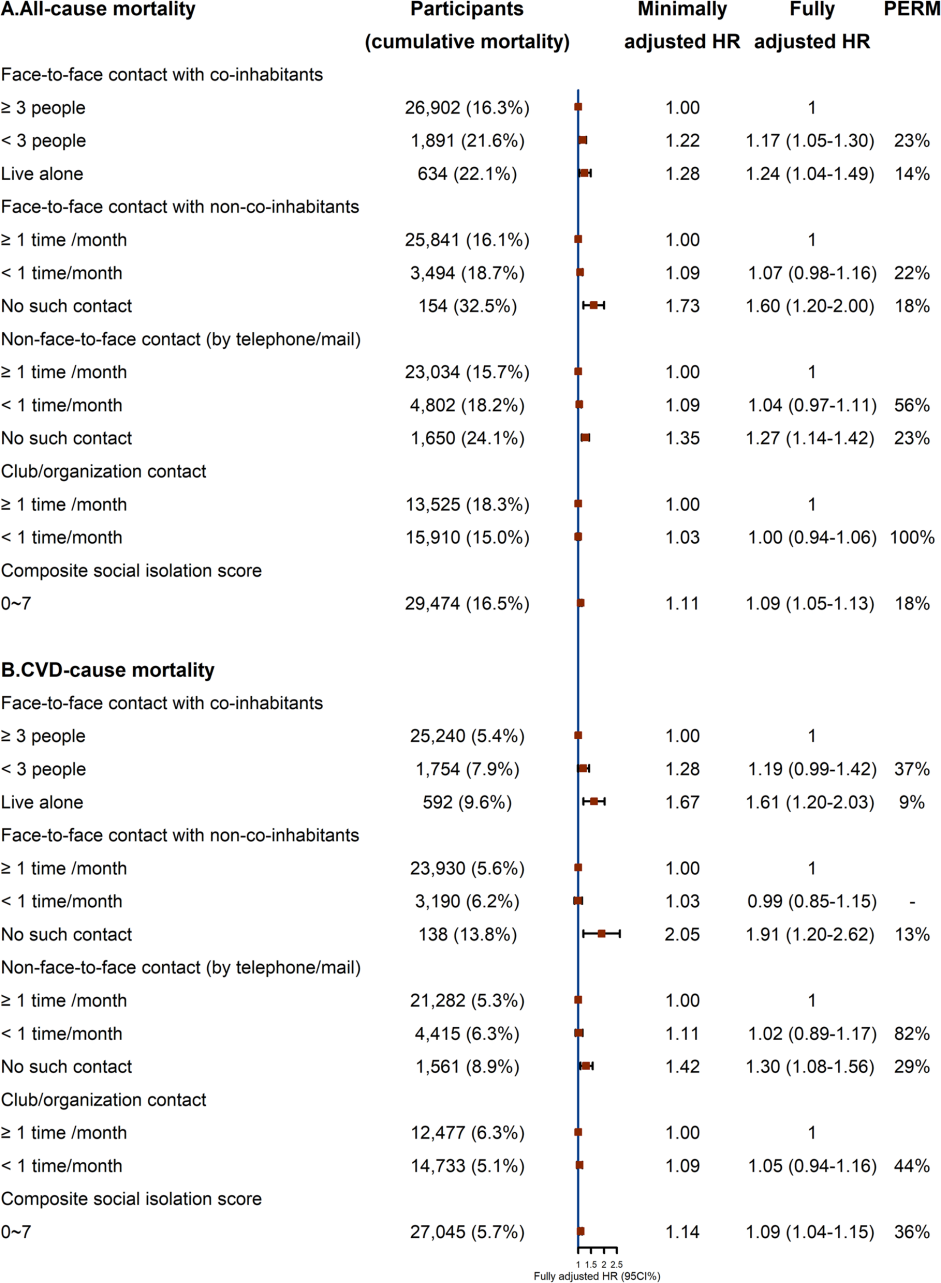
Associations of four types of social contact isolation with all-cause and cardiovascular disease mortality by Cox proportional hazards regession. HR, hazard ratio; CI, confidence interval; PERM, percentage of excess risk mediated; minimally adjusted HR: adjusted for sex, age, and self-rated health. Fully adjusted HR: adjusted for sex, age, self-rated health, socioeconomic position (education, occupation and family income), biological factors (body mass index, systolic blood pressure, diastolic blood pressure and fasting glucose), and behavioral factors (smoking status, alcohol use and physical activity)

**Fig. 4:**
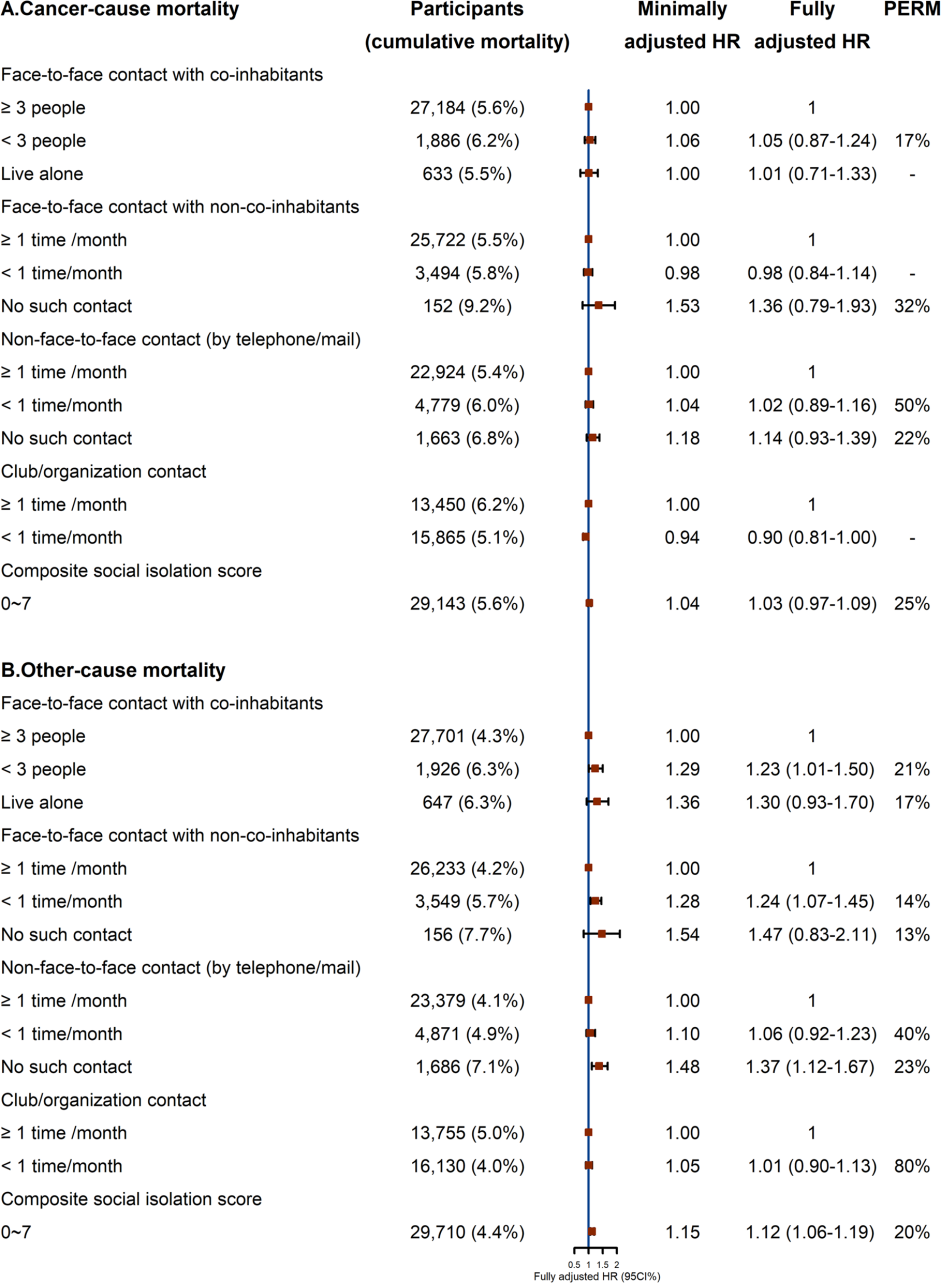
Associations of four types of social contact isolation with cancer and other-cause mortality by Cox proportional hazards regression. HR, hazard ratio; CI, confidence interval; PERM, percentage of excess risk mediated; minimally adjusted HR: adjusted for sex, age, and self-rated health. Fully adjusted HR: adjusted for sex, age, self-rated health, socioeconomic position (education, occupation and family income), biological factors (body mass index, systolic blood pressure, diastolic blood pressure and fasting glucose), and behavioral factors (smoking status, alcohol use and physical activity)

Figures 3 and 4 show the minimal adjust HR and fully adjusted HR with 95% CI of four types of social isolation with all-cause and cause-specific mortality. In Fig. 3, participants who lived alone had higher risks of all-cause (adjusted hazard ratio (AHR) = 1.24; 95% CI 1.04 to 1.49, *P* for trend < 0.001) and CVD (1.61;1.20–2.03, *P* for trend = 0.001) mortality than those who had ≥ 3 co-inhabitant contact. Compared with those who had ≥ 1 time/month non-co-inhabitant contact, those without such contact had higher risks of all-cause (1.60; 1.20–2.00, *P* for trend = 0.008) and CVD (1.91;1.20–2.62, *P* for trend = 0.008) mortality. The corresponding AHR (95% CI, *P* for trend) in participants without telephone/mail contact were 1.27 (1.14–1.42, < 0.001) and 1.30 (1.08–1.56, 0.009). In Fig. 4, no association of all four types of isolation with cancer mortality was found, but “< 3 people” with co-inhabitants (1.23; 1.01–1.50, *P* for trend = 0.026) and “< 1 time/month” with non-co-inhabitants (1.24; 1.07–1.45, *P* for trend = 0.003) of face-to-face contact was associated with higher other-cause mortality. No telephone/mail contact was also associated with higher other-cause mortality (1.37, 1.12–1.67, *P* for trend = 0.006). Furthermore, an increase in one composite social isolation score was associated with 9%, 9%, and 12% higher risk of all-cause, CVD-, and other-cause mortality respectively, with PERM showing 18%, 36%, and 20% risk explained by the thirteen factors. However, isolation from club/organization contact was not associated with the above mortality. The competing risk analyses (Additional file 1: Table S3) shows similar results as those from the main analysis using traditional Cox regression (Figs. 3 and 4).

In sensitivity analyses of the subsample of 19,947 participants, AHR for all-cause, CVD-, and other-cause mortality after additional adjustment for psychological factors in fully adjusted model become higher in those who lived alone, but attenuated for face-to-face with non-co-inhabitants and composite social isolation score (Additional file 1: Table S4). Almost all *P* values for test of interaction by sex, age, education, and health status on the associations of mortality and social isolation were not significant, and the few with *P* < 0.05 have become non-significant if multiple testing were accounted for (Additional file 1: Table S5-8). The face-to-face contact with co-inhabitants, non-co-inhabitants, and non-face-to-face contact remained positively associated with all-cause mortality after mutual adjustment, although the estimates were slightly attenuated (Additional file 1: Table S9). There are only slightly change before and after death within the first 2 years (Additional file 1: Table S10 compared with Fig. 3 which have excluded 244 deaths within first 2 years). The Kaplan-Meier survival curves show that the more severe social isolation was associated with lower survival probability in both baseline (2003–2008) and repeated (2008–2012) examination (Additional file 1: Fig S1 and S2).

## Discussion

### Principal findings

We have first, in a long-term prospective study (with about 13 years follow-up), show the positive associations of face-to-face and non-face-to-face isolation and a composite isolation score, with all-cause, CVD- and other-cause (but not cancer) mortality. Only 18–36% of the excess risk was attributable to the known risk factors such as SEP, biological, and behavioral factors. No association of club/organization contact (< 1 time/month versus ≥ 1 time/month) with all-cause and cause-specific mortality was found.

### Comparison with other studies

Our findings are in line with previous studies showing social isolation was associated with a higher risk of all-cause and CVD mortality [8, 13, 24, 32]. However, no previous studies reported the associations with face-to-face isolation and non-face-to-face isolation separately. For example, the UK Biobank study [32] assessed social isolation using a social isolation index, which only included questions on face-to-face contact. The Million Women Study [3] and English Longitudinal Study of Ageing [54] included results of a composite score with e-mail, phone and face-to-face contact but did not report results on different isolation types. The Whitehall II Study [55], the Nurses’ Health Study [56], and the Heinz Nixdorf Recall Study [24] also showed a higher mortality risk related to social isolation but did not specify the types of contact. Therefore, our study has provided additional evidence that both face-to-face and non-face-to-face isolation are associated with increased mortality risk. Our results on non-face-to-face contact are also consistent with a prospective study showing that participants with online social media experience (Facebook use) had a lower risk of mortality during two years follow-up than those without [57]. However, this study did not report the association of face-to-face contact with mortality, and the association of non-face-to-face contact was not adjusted for SEP and behavioral factors.

Furthermore, whether the associations of social isolation with mortality varied by sex, age, education, and health status is inconclusive [6, 18, 32, 54]. We found consistent associations of social isolation with all-cause, CVD-, and other-cause mortality after adjustment for sex, age, education, and health status. Our sex-stratified results are not consistent with one previous study showing the stronger associations of social isolation with CVD in women than men [32], neither consistent with other two reported stronger associations in men [24, 58]. We found the education did not modify the associations of social isolation with mortality, which was not consistent with previous studies showing the mortality risk of social isolation change depend upon SEP but without clarifying the types of social contact [27, 59, 60]. Moreover, living alone was particularly increased the CVD mortality in unhealthy participants suggesting the importance of immediate help among people who live alone, as a recent study reported [3].

Socially integrated people usually have more access to resources for health-promoting behavioral and chronic disease management [61]. Our results suggest that 18–36% of the excess all-cause, CVD-, and other-cause mortality risks could be attributable to SEP, biological, and behavioral factors, which is lower than those reported in two previous studies [5, 32], which showed that 64% and 84% mortality risk were explained by confounders or mediators. The fraction of excess risk remaining unexplained indicates some biological mechanisms beyond traditional pathways. For example, individuals with greater social support might have better immune function [62, 63]. Other pathways such as neuroendocrine mechanisms might also play a role [64, 65]. A recent genome-wide association study (GWAS) identified 38 significant genetic variants for social interaction, highlighting the possible genetic basis for social isolation [66]. Furthermore, another study found that gene expression differed between socially isolated and non-isolated individuals [67].

### Strengths and limitations

A major strength of the current study was that we, for the first time, analyzed the risk of mortality related to four important social isolation types: face-to-face isolation from co-inhabitants, face-to-face isolation from non-co-inhabitants, non-face-to-face isolation, and club/organization isolation, and provided the independent associations of face-to-face isolation and non-face-to-face isolation after mutual adjustment, which have never been reported in previous studies. The comprehensive analyses of four aspects of social isolation are essential because social contact patterns have been changing given the social-economic development. Our results demonstrated the beneficial role of non-face-to-face contact, which, if causal, would have important public health implications, although virtual contact has been the dominant form for non-face-to-face contact nowadays. Other strengths of our study included the long-term follow-up and the comprehensive adjustment for potential confounders. However, some limitations need to be considered. First, as about 98% of participants were retired at the time of baseline examination, workplace contact was not included in our study. Although the absence of workplace contact was unlikely to affect the internal validity of the present study, generalizability of our results to younger populations, in whom workplace contact may represent a major mode of contact, might be limited. Also, the other non-face-to-face contact such as instant message applications (video contact) and e-mail were not included. However, since this cohort study was initiated in 2003, such non-face-to-face contact was not prevalent, especially in older people. Specifically, the new technologies have been found to be effective in social contact for older people, but the findings were often mixed or inconclusive [68, 69]. Further studies are warranted to clarify. Second, we did not assess the effect of loneliness, which is a negative emotional state resulting from isolation, on mortality. However, isolation, which can be measured more objectively, rather than loneliness, was found to be an independent predictor of mortality [5]. Third, as evaluation of social isolation was based on self-report, reporting error was possible, although such error was more likely to be non-differential. Fourth, although residual confounding cannot be completely excluded in observational studies, the adjustment for a wide range of potential confounders in the current study should have minimized this bias. Fifth, reverse causality was possible, but we excluded deaths within 2 years in the main analysis and further conducted subgroup analyses in participants only with self-rated good/very good health status with no substantial changes in the original results. Sixth, we assessed social isolation at baseline and did not account for the changes in social isolation during the follow-up [70]. However, our sensitivity analysis of a subgroup of 18,104 participants who returned for repeated examination during 2008–2012 showed similar results to the main analyses (Additional file 1: Fig S1-2). Finally, as all GBCS participants were middle-aged and older Chinese, generalizability of our results to other populations or younger age groups may be limited.

## Conclusions

From a public health perspective, in the absence of well-designed trials of interventions to decrease social isolation with mortality as an outcome [71], our results suggest that people with face-to-face and non-face-to-face isolation both need more special attention and follow-up. Our findings, if causal, emphasize the importance of policies to test the effective way of increasing not only face-to-face but also non-face-to-face contact to promote physical and mental health.

## Supplementary Information

**Additional file 1: Table S1.** Summary information for studies about the associations of social isolation with all-cause mortality (from Jan 2015 to May 2021). **Table S2.** The missing percent of the variables in the cohort. **Table S3.** Associations of social isolation with cause-specific mortality using competing risk model (Fine-Gray’s model). **Table S4.** Associations of social isolation with all-cause and cause-specific mortality with additional adjustment for psychological factors† in the subgroup (*n*=19,947). **Table S5.** Associations of social isolation with all-cause and cause-specific mortality by sex (men, women) †. **Table S6.** Associations of social isolation with all-cause and cause-specific mortality by age group (< 60 years, ≥60 years) †. **Table S7.** Associations of social isolation with all-cause and cause-specific mortality by education group (≤ primary, ≥ middle school) †. **Table S8.** Associations of social isolation with all-cause and cause-specific mortality by health status (poor/very poor, good/very good) †. **Table S9.** Associations of face-to-face and non-face-to-face contact with all-cause mortality after mutual adjustment of 3 types of contact. **Table S10.** Associations of social isolation with all-cause mortality in original dataset (without excluding 244 death within first 2 years). **Figure S1.** Kaplan–Meier survival curve by four types of social isolation assessed at baseline examination (2003-2008) for all-cause mortality in 30,430 participants. **Figure S2.** Kaplan–Meier survival curve by four types of social isolation assessed at follow-up examination (2008-2012) for all-cause mortality in 18,104 participants.

## Data Availability

Ethical approval in place allows us to share data on requests. Please directly send such requests to the Guangzhou Biobank Cohort Study Data Access Committee (gbcsdata@hku.hk).

## Abbreviations

AHR: Adjusted hazard ratio
CVD: Cardiovascular disease
COVID: Coronavirus disease
GBCS: Guangzhou biobank cohort study
GHHARE: Guangzhou Health and Happiness Association for the Respectable Elders
CNY: Chinese yuan renminbi
US: United States
SNI: Social Network Index
ICD: International Classification of Diseases
SEP: Socioeconomic position
BMI: Body mass index
SBP: Systolic blood pressure
DBP: Diastolic blood pressure
PSS-14: Perceived Stress Scale-14 items
DWRT: Delayed Word Recall Test
PERM: Percentage of excess risk mediated
HR: Hazard ratios
CI: Confidence intervals
GWAS: Genome-wide association study
