# Supplementary material for: Associations of face-to-face and non-face-to-face social isolation with all-cause and cause-specific mortality: 13-year follow-up of the Guangzhou Biobank Cohort study
Source: BMC Med. 2022 May 2;20:178. doi: 10.1186/s12916-022-02368-3 (PMC9059436; doi:10.1186/s12916-022-02368-3)
Supplement: Supplementary file 1 — Additional file 1: Table S1. Summary information for studies about the associations of social isolation with all-cause mortality (from Jan 2015 to May 2021). Table S2. The missing percent of the variables in the cohort. Table S3. Associations of social isolation with cause-specific mortality using competing risk model (Fine-Gray’s model). Table S4. Associations of social isolation with all-cause and cause-specific mortality with additional adjustment for psychological factors† in the subgroup (n=19,947). Table S5. Associations of social isolation with all-cause and cause-specific mortality by sex (men, women) †. Table S6. Associations of social isolation with all-cause and cause-specific mortality by age group (< 60 years, ≥60 years) †. Table S7. Associations of social isolation with all-cause and cause-specific mortality by education group (≤ primary, ≥ middle school) †. Table S8. Associations of social isolation with all-cause and cause-specific mortality by health status (poor/very poor, good/very good) †. Table S9. Associations of face-to-face and non-face-to-face contact with all-cause mortality after mutual adjustment of 3 types of contact. Table S10. Associations of social isolation with all-cause mortality in original dataset (without excluding 244 death within first 2 years). Figure S1. Kaplan–Meier survival curve by four types of social isolation assessed at baseline examination (2003-2008) for all-cause mortality in 30,430 participants. Figure S2. Kaplan–Meier survival curve by four types of social isolation assessed at follow-up examination (2008-2012) for all-cause mortality in 18,104 participants. [file 12916_2022_2368_MOESM1_ESM.docx]

**Additional file:**

Table S1. Summary information for studies about the associations of social isolation with all-cause mortality (from Jan 2015 to May 2021).

Table S2. The missing percent of the variables in the cohort.

Table S3. Associations of social isolation with cause-specific mortality using competing risk model (Fine-Gray’s model).

Table S4. Associations of social isolation with all-cause and cause-specific mortality with additional adjustment for psychological factors^†^ in the subgroup (n=19,947).

Table S5. Associations of social isolation with all-cause and cause-specific mortality by sex (men, women) †.

Table S6. Associations of social isolation with all-cause and cause-specific mortality by age group (< 60 years, ≥60 years) †.

Table S7. Associations of social isolation with all-cause and cause-specific mortality by education group (≤ primary, ≥ middle school) †.

Table S8. Associations of social isolation with all-cause and cause-specific mortality by health status (poor/very poor, good/very good) †.

Table S9. Associations of face-to-face and non-face-to-face contact with all-cause mortality after mutual adjustment of 3 types of contact.

Table S10. Associations of social isolation with all-cause mortality in original dataset (without excluding 244 death within first 2 years).

Figure S1. Kaplan–Meier survival curve by four types of social isolation assessed at baseline examination (2003-2008) for all-cause mortality in 30,430 participants.

Figure S2. Kaplan–Meier survival curve by four types of social isolation assessed at follow-up examination (2008-2012) for all-cause mortality in 18,104 participants.

**Table S1. Summary information for studies about the associations of social isolation with all-cause mortality (from Jan 2015 to May 2021).**

|  | **Author** | **Follow-up time, years** | **Setting** | **Age, year** | **Sample size** | **Social isolation assessment** | **Main results** | **Confounders** |
| --- | --- | --- | --- | --- | --- | --- | --- | --- |
| 1 | Christopher | About 10 | US Health and Retirement Study | 50-95 | 11,302 | Social isolation: a score to each participant based on whether they 1) were unmarried, 2) lived alone, 3) had less than monthly contact with children, 4) had less than monthly contact with other family members, 5) had less than monthly contact with friends, and 6) did not participate monthly in any groups, clubs, or other social organizations, yielding scores 0-6. | Hazard ration (HR)=1.28, 95% confidence interval (CI) 1.05-1.56 | Socioeconomic and psychological risks |
| 2 | Kraav, S. L. | 23.2 | Finnish men | 42-61 | 2,588 | Social Isolation Scale consists of 10 items that measure the number of social contacts of the respondent. | HR=1.086, 95% CI: 1.024–1.153 | Age, year of examination, SES, alcohol consumption, smoking and physical activity, BSDS, hours slept at night, HPL depression score, hs-CRP, low-density lipoprotein cholesterol, history of ischemic heart disease, and systolic blood pressure |
| 3 | Lijun Fan | 10 | Chinese Longitudinal Healthy Longevity Survey | ≥65 | 11,934 | Social support was measured using a sum score of three dimensions (family support, social service and social security) with 22 items. | A higher sum score of social support at baseline reduced mortality risk (Adjust odds ratio (AOR) = 0.947, 95% CI:0.917-0.977). | Age, gender, residence (rural, urban), education attainment, main occupation, BMI, smoking and drinking. |
| 4 | Ryo Naito | 9 | 20 high-income, middle-income and low-income countries | 35-70 | 119,894 | Berkman and Syme constructed their social network index (5-items). The social isolation scale ranges from 0 to 5. | HR= 1.26, 95% CI: 1.17 - 1.36 | Age, sex, education, residence area, country income level, smoking, alcohol, hypertension, diabetes, coronary artery disease and stroke. COPD, chronic obstructive pulmonary disease. |
| 5 | Anna Uhing | About 10 | United States (MIDUS) survey | 20-75 | 6,259 | In self-administered questionnaires, participants were asked to rate their relationship with family(4-items), friends(4-items), and their spouse/partner(6-items) | HR from 0.81-1.13 | Sex, age group, race, education level, marital status, total household income. |
| 6 | Mark Ward | 7 | Irish Longitudinal Study | ≥50 | 6,915 | The size of social networks was measured using the Berkman-Syme Social Network Index | HR = 1.37, 95% CI: 1.04–1.81 | Sociodemographic and health-related covariates |
| 7 | Masashige Saito | 10 | Japan Gerontological Evaluation Study (JAGES) and the English Longitudinal Study of Ageing (ELSA). | JAGES ≥65;  ELSA ≥50. | 15,313 from JAGES 5,124 from ELSA | Social isolation was measured by two scales, i.e., scoring the frequency of contact with close ties, and a composite measurement of social isolation risk | JAGES: HR = 1.30, 95% CI: 1.12–1.50, in ELSA: HR = 2.05, 95% CI: 1.52–2.73. | Sex and age of the participants, self‐rated health, presence of medical treatment for existing health conditions, basic activities of daily living (ADL), marital status and household equivalized income |
| 8 | Tsutomu Uzuki | 5 | Japan | 40-74 | 16,651 | Social support was evaluated using five-component questions: Do you have someone 1) whom you can consult when you are in trouble? 2) whom you can consult when your physical condition is not good? 3) who can help you with daily homework? 4) who can take you to hospital when you don't feel well? and 5) who can take care of you when you are ill in bed? | HR = 2.01, 95% CI 1.26–3.05 | Age, gender, education period, smoking, alcohol consumption, obesity, hypertension, diabetes, and dyslipidemia. |
| 9 | Gilmour H | 8-9 | Canadian Community Health Survey-Healthy Aging data (2008/2009) | ≥65 | 13,037 | Respondents were asked how often in the past 12 months (at least once a day/week/month/year or never) they participated in each activity. Individuals whose overall participation was less than weekly were classified as having low participation versus high participation (one activity or more on a daily or weekly basis).(8-items) | Adjust hazard ratio (AHR)=1.3 95%CI 1.1-1.5 in men and AHR=1.4 95%CI 1.2-1.6 in women | Subjective isolation, socio-demographic characteristics, health status, and health behaviors. |
| 10 | Janine gronewold | 13.4 | Heinz Nixdorf recall study | 59.1 | 4,139 | We assessed social integration with the social integration index developed by Berkman et al.10 The index includes three types of ties: (1) marital Status/cohabitation, (2) contacts with close friends/family and (3) affiliation with voluntary associations. These types of ties each scored from 0 to 2, thus the index ranged from 0 to 6 | HR=1.47, 95% CI 1.09-1.97. | Minimally adjusted (adjusting for age, sex and social integration or social support, respectively); Biological factors; Health behavior; Socioeconomic Depression. |
| 11 | Nawi Ng | 26 | Västerbotten County | 50~70 | 22,226 men and 23,390 women | They were also asked the number of people:(i) who they know and interact with; (ii) who can come to their house anytime and feel at home; and (iii) who were family or friends with whom they can speak their mind openly | AHR = 1.38, 95% CI 1.26–1.50 in men and AHR=1.27, 95% CI 1.13–1.42 in women | Sociodemographic variables, as well as social capital and risk factor burden |
| 12 | Claudia Trudel-Fitzgerald | 18.73 | Nurses’ Health Study | 58.8 | 72,322 | Berkman–Syme SNI, administered via self-reported scale in 1992. The SNI assesses quantity and type of social relationships across four domains: marriage, contacts with close friends and relatives, participation in religious activities, and participation in group associations. | AOR 1.41, 95% CI 1.28–1.54 | Demographics and chronic diseases |
| 13 | Jinke Tan | 13.8 | 2001 US National Health Interview Survey (NHIS) | 18-85+ | 33,326 | Participants’ social support was assessed by the question “how often do you get the social and emotional support you need? Always, usually, sometimes, rarely, never.” The rarely and never categories were combined for analysis due to small frequencies. Rarely/never, sometimes, usually, always are scored on a scale from 1 to 4. The composite social integration was created and based on eight binary questions | HRs ranged from 0.94 to 0.70 | Age, sex, race, education level, income level, employment status, social support, BMI, smoking status, alcohol intake, history of hypertension, history of diabetes, history of heart disease, history of stroke, and history of cancer. |
| 14 | Nancy Freeborne | 10.8 | Women’s Health Initiative Observational Study in US | 50-79 | 92,715 women | Nine support items that assessed perception of functional support were selected from the 19-item Medical Outcomes Study Social Support Survey (MOS-SSS). | AHR= 0.95, 95% CI, 0.91-0.98 | Sociodemographic characteristics and select traditional CVD risk factors |
| 15 | Hirokazu Tanaka | 10 | Komo-Ise cohort study | 40-69 | 9533 | The social relationship score consisted of six items: (1) marital status, (2) living alone, (3) degree of social support, (4) participation in social activity, (5) social isolation, and (6) being bullied. | HR=1.44, 95% CI 1.06–1.90 | Age (5-year age categories), region of residence, smoking habits, drinking habits, and perceived health |
| 16 | Ryota Sakurai | 6 | Tokyo | 65+ | 1,023 | Participants were asked about the frequency of both face-to-face and non-face-to-face (e.g. telephone, e-mail, letters) interactions with family members who did not live with them, relatives, friends, and neighbors. The responses were then categorized as (1) every day, (2) 4–5 times a week, (3) 2–3 times a week, (4) once a week, (5) 2–3 times a month, (6) once a month, (7) less than once a month, or (8) no contact. | AHR= 2.19; 95% CI: 1.04–4.63 | Age, sex, number of years of education, comorbidities, depression symptoms, subjective health, and residential areas |
| 17 | Kristina Laugesen | 7 | Denmark | 25-79 | 31,500 | The four dichotomized characteristics summarized by the SNI are marriage/partnership; frequency of social contacts; frequency of religious participation; and memberships in groups (e.g., clubs, associations). | Adjusted mortality rate ratios (MRRs) =1.7, 95% CI:1.1–2.6 among men and 1.6, 95% CI: 0.83–2.9 among women. | Psychiatric and somatic status, lifestyle, and socioeconomic status |
| 18 | Christian Hakulinen | 7.1 | UK Biobank | 56+ | 479,054 | The social isolation scale contained three questions ((1) “Including yourself, how many people are living together in your household?”; (2) “How often do you visit friends or family or have them visit you?”; and (3) “Which of the following (leisure/social activities) do you engage in once a week or more often? You may select more than one”) | HR=1.25, 95% CI 1.03-1.51 | (1) biological (BMI, diastolic and systolic blood pressure, grip strength); (2) behavioural (alcohol consumption, physical activity and smoking); (3) socioeconomic (education, household income and Townsend Deprivation Index) and (4) mental health (depressive symptoms); and (5) history of chronic illness |
| 19 | Samuel G Smith | 7.7 | English Longitudinal Study of Ageing | ≥50 | 7,731 | Social isolation was defined according to marital/cohabiting status and contact with children, relatives, and friends, and participation in social organizations. Scores were split at the median to indicate social isolation (yes vs. no). | HR = 1.28, 95% CI 1.10-1.50 | Sociodemographic factors, health status, health behaviors, and cognitive function. |
| 20 | Kassandra I Alcaraz | 15 | American Cancer Society volunteers | 30+ | 1,185,106 | Social Network Index, the five-point social isolation score (exposure) was the sum of the four components, and ranged from 0 to 4 for least to most isolated | HR=2.34, 95%CI (1.58-3.46) and 1.60 (1.41-1.82) among black and white men, and 2.13 (1.44-3.15) and 1.84 (1.68-2.01) among black and white women. | Education, body mass index (Body Mass Index (BMI); smoking history and diabetes, and sex |
| 21 | Janet MacNeil-Vroomen | 1989-1990 | Cardiovascular Health Study | 65-80+ | 5,201 | Five items from the 6-item version of the Interpersonal Support Evaluation List (ISEL) were used to assess the perceived emotional, informational, and instrumental support. | Time-varying as well as baseline-only perceived social support were associated with greater survival in the unadjusted models but not after adjustment. | Demographic variables, stressful life events, depressive symptoms, the presence of major chronic conditions, subclinical cardiovascular disease, congestive heart failure status, coronary heart disease status, cognitive impairment. |
| 22 | Maarit Kauppi | 16 | Finnish Public Sector Study (FPS) and the Health and Social Support Study (HeSSup) | 42.9 and  36.7 | 7,617 and  20,816 | Social network size was assessed in both cohort studies at baseline using the social convoy model described by Antonucci. The model is based on a set of 3 concentric circles, each of which is considered to represent different levels of closeness to the respondent. | AHR = 1.23, 95% CI: 1.04-1.46 | Education; diagnosed chronic conditions, obesity, heavy alcohol consumption, smoking, and low physical activity |
| 23 | Beatriz Olaya | 3 | Spain | 60+ | 2,113 | The Oslo social support scale was used to assess social support. It has three items: “How many people are you so close to that you can count on them if you have great personal problems?”;“How much interest and concern do people show in what you do?” and “How easy is it to get practical help from neighbors if you should need it?”. A composite score was calculated as the sum of the three items, ranging from 3 to 14. | HR = 2.43, 95%CI = 1.14–5.18 | Age, gender, years of education, marital status, level of loneliness, tobacco and alcohol consumption, depression and memory function |
| 24 | Tábatta Renata Pereira de Brito | 3.9 | Brazil,2006 SABE Study | 60+ | 1,413 | The social network was evaluated using the variables: social support received; social support offered; number of members in the social network. | Having networks composed of 9 or more members reduced the risk of death in the older adults. | Age, gender, living arrangements, marital status, income, education, comorbidity, depressive symptoms, cognition and functional difficulties. |
| 25 | Marko Elovainio | 6.5 | UK Biobank | 56.5+ | 466,901 | 1) “Including yourself, how many people are living together in your household? Include those who usually live in the house such as students living away from home during term time, partners in the armed forces or professions such as pilots” (1 point for living alone); (2) “How often do you visit friends or family or have them visit you?” (1 point for friends and family visit less than once a month); and (3) “Which of the following [leisure/social activities] do you engage in once a week or more often? You may select more than one” (1 point for no participation in social activities at least weekly). Thus, individuals could score a total of 0–3; | AHR=1·26, 95% CI 1·20–1·33 | Biological (body-mass index, systolic and diastolic blood pressure, and handgrip strength), behavioural (smoking, alcohol consumption, and physical activity), socioeconomic (education, neighborhood deprivation, and household income), and psychological (depressive symptoms and cognitive capacity) risk factors. |
| 26 | Andrea Fleisch Marcus | 12-18 | Third National Health and Nutrition Examination Survey  (NHANES III) | 17-80+ | 20,024 | Social Network Index (SNI) that captures the four domains first assessed by Berkman and Syme. A total score, ranging from 0 to 4, was created by summing the four items. | HR=1.42, 95% (CI): 1.28-1.59 | Age, sex, race/ethnicity, and individual SEP |
| 27 | Terrence D Hill | 17 | Mexican Americans | 65+ | 2,334 | Social support including marital status, living arrangements, monthly contact with family and friends, religious attendance, and secular group memberships | HR =1.70 | Education, income, disengagement, support, or alcohol consumption |
| 28 | Katie M. Becofsky | 13.5 | Aerobics Center Longitudinal Study | 53 | 12,709 | (“Do you receive support from relatives and friends? Social support can be instrumental or emotional. Instrumental Support includes financial aid, information, help with family or work, advice, food, or transportation. Emotional Support includes affection, sympathy, trust, encouragement, or guidance. Please indicate whether or not you receive social support from each of the groups listed [Spouse or Partner, Relatives, Friends, and Overall relationships] by circling NO or YES.”). | Receiving social support from relatives: HR 0.81, 95% CI 0.68–0.95. Receiving spousal/partner support: HR 0.81, 95% CI 0.66-.99. Receiving social support from friends: HR 0.90, 95% CI 0.75–1.09, participants reporting social contact with 6 or 7 friends on a weekly basis: HR 0.76, 95% CI 0.58–0.98. | Age, gender, BMI (model 1), plus current smoking (yes/no), heavy alcohol intake (yes/no), and physical inactivity (yes/no) (model 2), plus presence of hypertension, high serum cholesterol, and diabetes at baseline (model 3) |
| 29 | Ziggi Ivan Santini | 3.8 | 8 countries (Cuba, Dominican Republic, Peru, Venezuela, Mexico, Puerto Rico, China, and India) | 65+ | 13,891 | Five network types were based on the participants' responses to eight questions: Distance to nearest relative (not spouse). Distance to nearest child. Distance to nearest sibling. Contact frequency with children or other relatives. Contact frequency with friends in the community/neighbourhood. Contact frequency with neighbours. Religious involvement. Involvement in community or social groups. | HR from 1.13-1.45 | Age, gender, marital status, education, number of assets, receipt of pension, disability, number of physical impairments, and depression |
| 30 | Tarja Nieminen | 9 | Finnish adult | 30-79 | 7,210 | Our data included altogether 36 variables indicating social capital that were selected based on previous literature. Based on explorative factor analysis, these variables formulated three different dimensions of social capital: (i) social support, (ii) social participation and networks and (iii) trust and reciprocity | HR=1.56, 95% CI 1.07–2.29 in men and HR=1.42 95% CI 0.95–2.12 in women | Socio-demographic factors, behavioural factors, biological risk factors, health status. |

*We only included the studies conducted in general population.

**Table S2. The missing percent of the variables in the cohort.**

| **Variables** | **Available number** | **Missing percent*** |
| --- | --- | --- |
| **Exposures** |  |  |
| Face-to-face contact with co-inhabitants | 30,510 | 0.03% |
| Face-to-face contact with non-co-inhabitants | 30,180 | 1.11% |
| Non-face-to-face contact (by telephone/mail) | 30,177 | 1.12% |
| Club/organization contact | 30,126 | 1.28% |
| **Outcome** |  |  |
| Mortality information | 30,143 | 1.23% |
| **Confounders** |  |  |
| Sex | 30,518 | 0.00% |
| Age | 30,518 | 0.00% |
| Self-rated health | 29,625 | 2.93% |
| Education | 30,496 | 0.07% |
| Occupation | 30,348 | 0.56% |
| Family income | 30,469 | 0.16% |
| BMI | 30,415 | 0.34% |
| SBP | 30,427 | 0.30% |
| DBP | 30,427 | 0.30% |
| FG | 30,335 | 0.60% |
| Smoking status | 30,413 | 0.34% |
| Alcohol | 30,289 | 0.75% |
| Physical activity | 30,110 | 1.34% |
| Cognitive function | 29,983 | 1.75% |

BMI=body mass index; SBP=systolic blood pressure; DBP=diastolic blood pressure; FG=fasting glucose.

* Proportion of missing was the number of participants who did not provide the related information divided by the total number of GBCS participants (n=30,518).

**Table S3. Associations of social isolation with cause-specific mortality using competing risk model (Fine-Gray’s model).**

| **Social contact isolation types** | **Number/frequency** | **CVD mortality** | **Cancer mortality** | **Other-cause mortality** |
| --- | --- | --- | --- | --- |
|  |  | **SHR (95% CI)^†^** | **SHR (95% CI)^†^** | **SHR (95% CI)^†^** |
| Face-to-face contact with co-inhabitants | ≥3 people | 1.00 | 1.00 | 1.00 |
|  | < 3 people | 1.15 (0.91-1.43) | 1.02 (0.86-1.21) | 1.25 (1.09-1.53) * |
|  | Live alone | 1.50 (1.02-2.22) ** | 0.91 (0.67-1.25) | 1.27 (0.90-1.75) |
| Face-to-face contact with non-co-inhabitants | ≥1 time /month | 1.00 | 1.00 | 1.00 |
|  | < 1 time/month | 1.07 (0.72-1.06) | 0.98 (0.86-1.13) | 1.29 (1.13-1.49) ** |
|  | No such contact | 1.54 (1.07-2.03) ** | 1.35 (0.84-2.29) | 1.71 (1.08-2.70) * |
| Non-face-to-face contact (by telephone/mail) | ≥1 time /month | 1.00 | 1.00 | 1.00 |
|  | < 1 time/month | 1.03 (0.81-1.30) | 1.04 (0.93-1.17) | 1.07 (0.95-1.22) |
|  | No such contact | 1.29 (1.08-1.54) ** | 1.03 (0.86-1.24) | 1.29 (1.08-1.54) ** |
| Club/organization contact | ≥1 time /month | 1.00 | 1.00 | 1.00 |
|  | < 1 time/month | 1.04 (0.87-1.24) | 0.95 (0.87-1.04) | 1.02 (0.94-1.14) |
| Composite social isolation score^#^ | 0~7 | 1.09 (1.05-1.15) ** | 1.02 (0.97-1.07) | 1.12 (1.07-1.18) ** |

SHR= subdistribution hazard ratio; CI=confidence interval.

^#^ Social isolation score was treated as a continuous variable.

^†^ Adjusted for sex, age, self-rated health, socioeconomic position, biological factors (body mass index, systolic blood pressure, diastolic blood pressure and fasting glucose) and behavioral factors (smoking status, alcohol use and physical activity).

* P<0.05; ** P<0.001.

**Table S4. Associations of social isolation with all-cause and cause-specific mortality with additional adjustment for psychological factors^†^ in the subgroup (n=19,947).**

| **Social contact isolation types** | **Number/frequency** | **Participants/deaths** | **All-cause**  **mortality** | **Participants/**  **deaths** | **CVD mortality** | **Participants/deaths** | **Cancer mortality** | **Participants/deaths** | **Other-cause**  **mortality** |
| --- | --- | --- | --- | --- | --- | --- | --- | --- | --- |
|  |  |  | **HR; 95% CI** |  | **HR; 95% CI** |  | **HR; 95% CI** |  | **HR; 95% CI** |
| Face-to-face contact with  co-inhabitants | ≥3 people | 17,964/3,265 | 1.00 | 16,588/1,011 | 1.00 | 17,897/1,111 | 1.00 | 18,261/894 | 1.00 |
|  | < 3 people | 812/247 | 1.18 (1.03-1.35) * | 758/86 | 1.14 (0.91-1.43) | 8/62 | 1.04 (0.80-1.35) | 833/78 | 1.12 (1.00-1.61) * |
|  | Live alone | 101/37 | 1.38 (1.00-1.92) * | 97/16 | 1.78 (1.08-2.92) * | 101/8 | 1.05 (0.52-2.11) | 104/13 | 1.62 (0.93-2.80) |
| Face-to-face contact with  non-co-inhabitants | ≥1 time /month | 16,776/3,097 | 1.00 | 15,517/982 | 1.00 | 16,701/1,047 | 1.00 | 17,056/839 | 1.00 |
|  | < 1 time/month | 2,032/428 | 1.01 (0.91-1.12) | 1,860/121 | 0.87 (0.72-1.06) | 2,040/128 | 0.93 (0.77-1.12) | 2,070/139 | 1.20 (1.00-1.43) * |
|  | No such contact | 57/20 | 1.22 (0.77-1.95) | 53/9 | 1.47 (0.73-2.97) | 58/5 | 0.90 (0.33-2.40) | 59/5 | 1.13 (0.47-2.73) |
| Non-face-to-face contact  (by telephone/mail) | ≥1 time /month | 15,105/2,731 | 1.00 | 13,974/848 | 1.00 | 15,041/924 | 1.00 | 15,357/762 | 1.00 |
|  | < 1 time/month | 2,845/572 | 1.03 (0.94-1.12) | 2,591/184 | 1.00 (0.98-1.01) | 2,829/185 | 0.99 (0.84-1.16) | 2,889/149 | 0.96 (0.81-1.15) |
|  | No such contact | 918/242 | 1.27 (1.11-1.45) ** | 870/79 | 1.05 (0.93-1.19) | 933/72 | 1.20 (0.94-1.53) | 943/72 | 1.29 (1.00-1.65) * |
| Club/organization contact | ≥1 time /month | 9,464/1,955 | 1.00 | 8,739/611 | 1.00 | 9,418/665 | 1.00 | 9,636/549 | 1.00 |
|  | < 1 time/month | 9,387/1,589 | 0.99 (0.92-1.06) | 8,678/500 | 1.05 (0.93-1.19) | 9,368/516 | 0.86 (0.76-0.97) * | 9,536/433 | 1.00 (0.87-1.13) |
| Composite social isolation score^#^ | 0~7 | 18,838/3,539 | 1.07 (1.03-1.11) * | 117,404/1,109 | 1.05 (0.98-1.13) | 18,772/1,180 | 1.02 (0.94-1.10) | 19,158/980 | 1.10 (1.02-1.18) * |

* P<0.05; ** P<0.001.

HR=hazard ratio; CI=confidence interval.

^#^ Social isolation score was calculated as a continuous variable.

† Factors adjusted including sex, age, self-rated health, socioeconomic position, biological factors (body mass index, systolic blood pressure, diastolic blood pressure and fasting glucose), behavioral factors (smoking status, alcohol use and physical activity) and psychological factors (stress level and cognitive function).

**Table S5. Associations of social isolation with all-cause and cause-specific mortality by sex (men, women) ^†^.**

| **Sex** | **Social contact isolation types** | **Number/frequency** | **Participants/**  **deaths** | **All-cause**  **mortality**  **HR; 95% CI** | **P for interaction** | **Participants/deaths** | **CVD mortality**  **HR; 95% CI** | **P for interaction** | **Participants/deaths** | **Cancer mortality**  **HR; 95% CI** | **P for interaction** | **Participants/deaths** | **Other-cause mortality**  **HR; 95% CI** | **P for interaction** |
| --- | --- | --- | --- | --- | --- | --- | --- | --- | --- | --- | --- | --- | --- | --- |
| Men (n=8,315) | Face-to-face contact with co-inhabitants | ≥3 people | 7,371/1,915 | 1.00 | 0.18 | 6,846/577 | 1.00 | 0.44 | 7,343/661 | 1.00 | 0.01 | 7,473/553 | 1.00 | 0.45 |
|  |  | < 3 people | 629/206 | 1.19 (1.02-1.38) * |  | 592/61 | 1.13 (0.86-1.48) |  | 627/67 | 1.15 (0.88-1.50) |  | 664/58 | 1.13 (0.85-1.50) |  |
|  |  | Live alone | 193/73 | 1.44 (1.12-1.85) * |  | 176/26 | 1.89 (1.24-2.90) * |  | 194/23 | 1.48 (0.96-2.29) |  | 198/21 | 1.33 (0.82-2.14) |  |
|  | Face-to-face contact with non-co-inhabitants | ≥1 time /month | 6,907/1,810 | 1.00 | 0.09 | 6,431/562 | 1.00 | 0.48 | 6,877/606 | 1.00 | 0.002 | 7,004/522 | 1.00 | 0.01 |
|  |  | < 1 time/month | 1,129/335 | 1.11 (0.99-1.25) |  | 1,034/87 | 0.96 (0.76-1.20) |  | 1,126/124 | 1.23 (1.00-1.50) |  | 1,150/100 | 1.14 (0.91-1.42) |  |
|  |  | No such contact | 56/20 | 1.12 (0.70-1.82) |  | 48/7 | 1.59 (0.75-3.37) |  | 56/8 | 1.34 (0.64-2.84) |  | 56/2 | 0.42 (0.11-1.70) |  |
|  | Non-face-to-face contact (by telephone/mail) | ≥1 time /month | 6,022/1,555 | 1.00 | 0.83 | 5,594/464 | 1.00 | 0.84 | 5,995/538 | 1.00 | 0.83 | 6,107/452 | 1.00 | 0.22 |
|  |  | < 1 time/month | 1,561/435 | 1.02 (0.91-1.13) |  | 1,447/132 | 0.98 (0.80-1.20) |  | 1,554/150 | 1.02 (0.84-1.23) |  | 1,584/123 | 1.02 (0.83-1.25) |  |
|  |  | No such contact | 507/174 | 1.20 (1.01-1.41) * |  | 472/59 | 1.35 (1.02-1.79) * |  | 508/51 | 1.02 (0.75-1.39) |  | 517/49 | 1.12 (0.82-1.53) |  |
|  | Club/organization contact | ≥1 time /month | 3,340/1,002 | 1.00 | 0.14 | 3,091/313 | 1.00 | 0.56 | 3,328/332 | 1.00 | 0.08 | 3,395/300 | 1.00 | 0.75 |
|  |  | < 1 time/month | 4,744/1,162 | 1.04 (0.95-1.14) |  | 4,416/343 | 1.00 (0.86-1.19) |  | 4,723/406 | 0.99 (0.85-1.15) |  | 4,807/323 | 1.05 (0.89-1.24) |  |
|  | Composite social isolation score^#^ | 0~7 | 8,070/2,157 | 1.07 (1.03-1.12) * | 0.79 | 7,495/653 | 1.08 (0.99-1.17) | 0.61 | 8,037/737 | 1.08 (1.00-1.16) | 0.44 | 8,188/621 | 1.05 (0.96-1.14) * | 0.42 |
| Women(n=21,959) | Face-to-face contact with co-inhabitants | ≥3 people | 19,915/2,469 | 1.00 |  | 18,394/792 | 1.00 |  | 19,845/849 | 1.00 |  | 20,228/625 | 1.00 |  |
|  |  | < 3 people | 1,262/203 | 1.16 (1.00-1.34) * |  | 1,162/78 | 1.25 (0.98-1.60) |  | 1,259/50 | 0.95 (0.71-1.28) |  | 1,282/63 | 1.35 (1.03-1.77) * |  |
|  |  | Live alone | 441/67 | 1.08 (0.83-1.40) |  | 416/31 | 1.43 (0.98-2.09) |  | 439/12 | 0.60 (0.32-1.12) |  | 449/20 | 1.25 (0.79-1.99) |  |
|  | Face-to-face contact with non-co-inhabitants | ≥1 time /month | 18,934/2,362 | 1.00 |  | 17,499/772 | 1.00 |  | 18,845/817 | 1.00 |  | 19,229/586 | 1.00 |  |
|  |  | < 1 time/month | 2,365/320 | 1.03 (0.91-1.16) |  | 2,156/110 | 1.02 (0.83-1.26) |  | 2,368/77 | 0.75 (0.59-0.95) * |  | 2,399/104 | 1.34 (1.09-1.66) * |  |
|  |  | No such contact | 98/30 | 2.08 (1.44-3.01) ** |  | 90/12 | 2.19 (1.20-3.99) ** |  | 96/6 | 1.34 (0.60-3.00) |  | 100/10 | 2.69 (1.43-5.06) ** |  |
|  | Non-face-to-face contact (by telephone/mail) | ≥1 time /month | 17,012/2,051 | 1.00 |  | 15,688/667 | 1.00 |  | 16,929/703 | 1.00 |  | 17,272/513 | 1.00 |  |
|  |  | < 1 time/month | 3,241/437 | 1.07 (0.96-1.19) |  | 2,968/147 | 1.07 (0.89-1.29) |  | 3,225/135 | 1.02 (0.84-1.23) |  | 3,287/116 | 1.11 (0.90-1.36) |  |
|  |  | No such contact | 1,143/224 | 1.32 (1.14-1.52) ** |  | 1,089/80 | 1.24 (0.98-1.58) |  | 1,155/62 | 1.22 (0.93-1.59) |  | 1,169/71 | 1.55 (1.20-2.01) ** |  |
|  | Club/organization contact | ≥1 time /month | 10,185/1,479 | 1.00 |  | 9,386/479 | 1.00 |  | 10,122/501 | 1.00 |  | 10,360/384 | 1.00 |  |
|  |  | < 1 time/month | 11,166/1,225 | 0.97 (0.89-1.05) |  | 10,317/412 | 1.07 (0.94-1.23) |  | 11,142/396 | 0.84 (0.73-0.97) * |  | 11,323/315 | 0.99 (0.84-1.15) |  |
|  | Composite social isolation score^#^ | 0~7 | 21,339/2,703 | 1.09 (1.04-1.13) * |  | 19,690/890 | 1.10 (1.03-1.18) * |  | 21,670/939 | 0.98 (0.90-1.06) |  | 21,670/699 | 1.19 (1.11-1.28) * |  |

* P<0.05; ** P<0.001. HR=hazard ratio; CI=confidence interval. ^#^ Social isolation score was calculated as a continuous variable. † Factors adjusted including age, self-rated health, socioeconomic position, biological factors and behavioral factors.

**Table S6. Associations of social isolation with all-cause and cause-specific mortality by age group (< 60 years, ≥60 years) ^†^.**

| **Age** | **Social contact isolation types** | **Number/frequency** | **Participants/**  **deaths** | **All-cause**  **mortality** | **P for interaction** | **Participants/**  **deaths** | **CVD mortality**  **HR; 95% CI** | **P for interaction** | **Participants/**  **deaths** | **Cancer mortality**  **HR; 95% CI** | **P for interaction** | **Participants/**  **deaths** | **Other-cause Mortality** | **P for interaction** |
| --- | --- | --- | --- | --- | --- | --- | --- | --- | --- | --- | --- | --- | --- | --- |
|  |  |  |  | **HR; 95% CI** |  |  |  |  |  |  |  |  | **HR; 95% CI** |  |
| < 60 years  (n=13,359) | Face-to-face contact with co-inhabitants | ≥3 people | 12,190/679 | 1.00 | 0.88 | 11,601/158 | 1.00 | 0.23 | 12,140/347 | 1.00 | 0.79 | 12,348/138 | 1.00 | 0.65 |
|  |  | < 3 people | 765/50 | 1.23 (0.90-1.66) |  | 727/13 | 1.48 (0.82-2.68) |  | 767/22 | 1.01 (0.63-1.61) |  | 778/12 | 1.55 (0.83-2.89) |  |
|  |  | Live alone | 231/17 | 1.28 (0.75-2.19) |  | 222/2 | 0.84 (0.21-3.43) |  | 228/8 | 1.38 (0.65-2.94) |  | 233/6 | 1.91 (0.76-4.77) |  |
|  | Face-to-face contact with non-co-inhabitants | ≥1 time /month | 11,631/649 | 1.00 | 0.72 | 11,084/155 | 1.00 | 0.62 | 11,582/328 | 1.00 | 0.91 | 11,784/134 | 1.00 | 0.25 |
|  |  | < 1 time/month | 1,326/76 | 0.96 (0.75-1.23) |  | 1,237/14 | 0.73 (0.41-1.28) |  | 1,318/38 | 1.03 (0.73-1.44) |  | 1,338/18 | 0.97 (0.57-1.66) |  |
|  |  | No such contact | 54/7 | 1.34 (0.55-3.25) |  | 49/3 | 2.25 (0.55-9.28) |  | 52/2 | 1.34 (0.33-5.45) |  | 54/1 | 0.90 (0.12-6.55) |  |
|  | Non-face-to-face contact (by telephone/mail) | ≥1 time /month | 10,467/568 | 1.00 | 0.63 | 9,973/142 | 1.00 | 0.12 | 10,416/273 | 1.00 | 0.14 | 10,598/123 | 1.00 | 0.29 |
|  |  | < 1 time/month | 1,946/115 | 0.97 (0.78-1.19) |  | 1,827/19 | 0.66 (0.40-1.08) |  | 1,938/68 | 1.21 (0.92-1.60) |  | 1,970/20 | 0.72 (0.44-1.19) |  |
|  |  | No such contact | 596/48 | 1.29 (0.95-1.76) |  | 568/10 | 1.13 (0.59-2.17) |  | 596/27 | 1.63 (1.08-2.45) * |  | 606/10 | 1.15 (0.58-2.28) |  |
|  | Club/organization contact | ≥1 time /month | 5,198/317 | 1.00 | 0.01 | 4,923/73 | 1.00 | 0.51 | 5,163/165 | 1.00 | 0.07 | 5,271/64 | 1.00 | 0.47 |
|  |  | < 1 time/month | 7,783/413 | 0.81 (0.70-0.95) * |  | 7,417/98 | 0.87 (0.63-1.21) |  | 7,759/202 | 0.76 (0.61-0.94) * |  | 7,875/89 | 0.84 (0.59-1.19) |  |
|  | Composite social isolation score^#^ | 0~7 | 12,975/730 | 1.06 (0.97-1.16) | 0.80 | 12,334/171 | 0.96 (0.79-1.18) | 0.002 | 12,916/367 | 1.13 (1.00-1.28) * | 0.57 | 13,171/153 | 1.05 (0.87-1.26) | 0.003 |
| ≥60 years  (n=16,915) | Face-to-face contact with co-inhabitants | ≥3 people | 15,096/3,705 | 1.00 |  | 13,639/1,211 | 1.00 |  | 15,044/1,163 | 1.00 |  | 15,353/1,040 | 1.00 |  |
|  |  | < 3 people | 1,126/359 | 1.17 (1.04-1.31) * |  | 1,027/126 | 1.17 (0.97-1.42) |  | 1,119/95 | 1.07 (0.86-1.32) |  | 1,148/109 | 1.21 (0.99-1.49) * |  |
|  |  | Live alone | 403/123 | 1.23 (1.01,1.49) * |  | 370/55 | 1.67 (1.25-2.22) ** |  | 405/27 | 0.94 (0.63-1.40) |  | 414/35 | 1.21 (0.85-1.73) |  |
|  | Face-to-face contact with non-co-inhabitants | ≥1 time /month | 14,210/3,523 | 1.00 |  | 12,846/1,179 | 1.00 |  | 14,140/1,095 | 1.00 |  | 14,449/974 | 1.00 |  |
|  |  | < 1 time/month | 2,168/579 | 1.09 (0.99-1.19) |  | 1,953/183 | 1.02 (0.87-1.19) |  | 2,176/163 | 0.97 (0.82-1.15) |  | 2,211/186 | 1.28 (1.09-1.50) * |  |
|  |  | No such contact | 100/43 | 1.63 (1.20-2.22) * |  | 89/16 | 1.89 (1.15-3.10) * |  | 100,12 | 1.38 (0.76-2.50) |  | 102/11 | 1.50 (0.83-2.72) |  |
|  | Non-face-to-face contact (by telephone/mail) | ≥1 time /month | 12,567/3,038 | 1.00 |  | 11,309/989 | 1.00 |  | 12,508/968 | 1.00 |  | 12,781/842 | 1.00 |  |
|  |  | < 1 time/month | 2,356/757 | 1.06 (0.98-1.15) |  | 2,588/260 | 1.08 (0.93-1.24) |  | 2,841/217 | 0.97 (0.83-1.13) |  | 2,901/219 | 1.11 (0.95-1.29) |  |
|  |  | No such contact | 1,054/350 | 1.28 (1.14-1.43) ** |  | 993/129 | 1.33 (1.10-1.60) ** |  | 1,067/86 | 1.04 (0.83-1.31) |  | 1,080/110 | 1.40 (1.14-1.73) ** |  |
|  | Club/organization contact | ≥1 time /month | 8,327/2,164 | 1.00 |  | 7,554/719 | 1.00 |  | 8,287/668 | 1.00 |  | 8,484/620 | 1.00 |  |
|  |  | < 1 time/month | 8,127/1,974 | 1.03 (0.96-1.10) |  | 7,316/657 | 1.07 (0.96-1.19) |  | 8,106/600 | 0.95 (0.84-1.06) |  | 8,255/549 | 1.02 (0.91-1.15) |  |
|  | Composite social isolation score^#^ | 0~7 | 16,434/4,130 | 1.08 (1.05-1.12) ** |  | 14,851/1,372 | 1.10 (1.04-1.17) * |  | 16,372/1,267 | 1.00 (0.94-1.07) |  | 16,752/1,170 | 1.13 (1.07-1.20) ** |  |

* P<0.05; ** P<0.001. HR=hazard ratio; CI=confidence interval. ^#^ Social score was calculated as a continuous variable. † Factors adjusted including sex, self-rated health, socioeconomic position, biological factors and behavioral factors.

**Table S7. Associations of social isolation with all-cause and cause-specific mortality by education group (≤primary, ≥middle school) ^†^.**

| **Education** | **Social contact isolation types** | **Number/frequency** | **Participants/**  **deaths** | **All-cause**  **mortality** | **P for interaction** | **Participants/deaths** | **CVD mortality**  **HR; 95% CI** | **P for interaction** | **Participants/deaths** | **Cancer mortality**  **HR; 95% CI** | **P for interaction** | **Participants/**  **deaths** | **Other-cause Mortality** | **P for interaction** |
| --- | --- | --- | --- | --- | --- | --- | --- | --- | --- | --- | --- | --- | --- | --- |
|  |  |  |  | **HR; 95% CI** |  |  |  |  |  |  |  |  | **HR; 95% CI** |  |
| ≤ primary  (n=12,963) | Face-to-face contact with co-inhabitants | ≥3 people | 11,468/2,337 | 1.00 | 0.11 | 10,642/791 | 1.0 | 0.20 | 11,470/717 | 1.00 | 0.26 | 11,651/659 | 1.00 | 0.55 |
|  |  | < 3 people | 908/243 | 1.20 (1.05-1.38) ** |  | 849/87 | 1.19 (0.94-1.50) * |  | 905/70 | 1.25 (0.97-1.61) |  | 923/71 | 1.19 (0.92-1.54) |  |
|  |  | Live alone | 380/93 | 1.20 (0.96-1.50) |  | 355/45 | 1.67 (1.21-2.30) ** |  | 383/19 | 0.82 (0.51-1.34) |  | 389/25 | 1.19 (0.78-1.82) |  |
|  | Face-to-face contact with non-co-inhabitants | ≥1 time /month | 10,877/2,230 | 1.00 | 0.07 | 10,117/776 | 1.00 | 0.62 | 10,874/679 | 1.00 | 0.50 | 11,057/616 | 1.00 | 0.25 |
|  |  | < 1 time/month | 1,668/383 | 1.05 (0.94-1.18) |  | 1,521/127 | 1.01 (0.83-1.22) |  | 1,668/109 | 0.97 (0.79-1.20) |  | 1,688/119 | 1.20 (0.98-1.47) |  |
|  |  | No such contact | 84/31 | 1.62 (1.13-2.33) ** |  | 78/13 | 1.83 (1.03-3.25) * |  | 85/6 | 1.02 (0.45-2.28) |  | 86/11 | 2.12 (1.16-3.87) * |  |
|  | Non-face-to-face contact (by telephone/mail) | ≥1 time /month | 9,249/1,828 | 1.00 | 0.23 | 8,558/618 | 1.00 | 0.36 | 9,242/561 | 1.00 | 0.82 | 9,401/514 | 1.00 | 0.80 |
|  |  | < 1 time/month | 2,330/522 | 1.05 (0.95-1.16) |  | 2,159/190 | 1.12 (0.94-1.32) |  | 2,324/151 | 1.00 (0.83-1.20) |  | 2,360/145 | 1.04 (0.86-1.26) |  |
|  |  | No such contact | 1,048/293 | 1.27 (1.12-1.44) ** |  | 998/107 | 1.31 (1.06-1.61) * |  | 1,060/82 | 1.18 (0.92-1.50) |  | 1,069/87 | 1.31 (1.03-1.66) * |  |
|  | Club/organization contact | ≥1 time /month | 5,613/1,283 | 1.00 | 0.18 | 5,197/436 | 1.00 | 0.10 | 5,607/390 | 1.00 | 0.91 | 5,712/372 | 1.00 | 0.47 |
|  |  | < 1 time/month | 7,000/1,355 | 1.01 (0.93-1.09) |  | 6,505/477 | 1.13 (0.99-1.30) |  | 7,005/403 | 0.86 (0.74-1.00) |  | 7,104/373 | 1.09 (1.05-1.13) * |  |
|  | Composite social isolation score^#^ | 0~7 | 12,600/2,633 | 1.08 (1.04-1.12) ** | 0.52 | 11,688/910 | 1.11 (1.04-1.18) * | 0.64 | 12,598/793 | 1.03 (0.96-1.11) | 0.80 | 12,802/1,069 | 1.11 (1.04-1.19) * | 0.16 |
| ≥ middle school  (n=17,289) | Face-to-face contact with co-inhabitants | ≥3 people | 15,800/2,040 | 1.00 |  | 14,577/575 | 1.00 |  | 15,693/791 | 1.00 |  | 16,028/518 | 1.00 |  |
|  |  | < 3 people | 983/166 | 1.12 (0.95-1.32) |  | 905/52 | 1.15 (0.86-1.55) |  | 981/47 | 0.86 (0.63-1.17) |  | 1,003/50 | 1.27 (0.94-1.71) |  |
|  |  | Live alone | 254/47 | 1.36 (0.99 -1.86) |  | 237/12 | 1.35 (0.74-2.46) |  | 250/16 | 1.38 (0.83-2.31) |  | 258/16 | 1.66 (0.97-2.84) |  |
|  | Face-to-face contact with non-co-inhabitants | ≥1 time /month | 14,956/1,939 | 1.00 |  | 13,805/557 | 1.00 |  | 14,841/743 | 1.00 |  | 15,168/492 | 1.00 |  |
|  |  | < 1 time/month | 1,824/272 | 1.08 (0.95-1.23) |  | 1,667/70 | 0.97 (0.75-1.25) |  | 1,824/92 | 0.99 (0.79-1.23) |  | 1,859/85 | 1.29 (1.02-1.63) * |  |
|  |  | No such contact | 69/18 | 1.57 (0.95-2.57) |  | 60/6 | 2.10 (0.94-4.73) |  | 66/7 | 1.90 (0.90-4.02) |  | 69/1 | 0.33 (0.05-2.37) |  |
|  | Non-face-to-face contact (by telephone/mail) | ≥1 time /month | 13,778/1,775 | 1.00 |  | 12,718/512 | 1.00 |  | 13,676/679 | 1.00 |  | 13,971/451 | 1.00 |  |
|  |  | < 1 time/month | 2,469/349 | 1.03 (0.91-1.16) |  | 2,253/89 | 0.88 (0.70-1.12) |  | 2,452/133 | 1.04 (0.86-1.26) |  | 2,508/94 | 1.08 (0.86-1.36) |  |
|  |  | No such contact | 601/105 | 1.29 (1.06-1.58) * |  | 562/32 | 1.34 (0.93-1.92) |  | 602/31 | 1.05 (0.73-1.51) |  | 616/33 | 1.57 (1.10-2.24) * |  |
|  | Club/organization contact | ≥1 time /month | 7,907/1,195 | 1.00 |  | 7,276/355 | 1.00 |  | 7,838/441 | 1.00 |  | 8,038/312 | 1.00 |  |
|  |  | < 1 time/month | 8,904/1,031 | 0.98 (0.90-1.07) |  | 8,222/278 | 0.93 (0.79-1.10) |  | 8,855/399 | 0.94 (0.81-1.08) |  | 9,020/265 | 1.03 (0.86-1.22) |  |
|  | Composite social isolation score^#^ | 0~7 | 16,798/2,223 | 1.08 (1.03-1.14) * |  | 15,487/632 | 1.05 (0.95-1.15) |  | 16,680/839 | 1.02 (0.94-1.11) |  | 17,045/766 | 1.15 (1.05-1.26) * |  |

* P<0.05; ** P<0.001. HR=hazard ratio; CI=confidence interval. ^#^ Social isolation score was calculated as a continuous variable. † Factors adjusted including sex, age, self-rated health, SEP (except education), biological and behavioral factors.

**Table S8. Associations of social isolation types with all-cause and cause-specific mortality by health status (poor/very poor, good/very good) †.**

| **Health status** | **Social contact isolation types** | **Number/frequency** | **Participants/**  **deaths** | **All-cause**  **mortality** | **P for interaction** | **Participants/**  **deaths** | **CVD mortality** | **P for interaction** | **Participants/**  **deaths** | **Cancer mortality** | **P for interaction** | **Participants/deaths** | **Other-cause Mortality** | **P for interaction** |
| --- | --- | --- | --- | --- | --- | --- | --- | --- | --- | --- | --- | --- | --- | --- |
|  |  |  |  |  |  |  |  |  |  |  |  |  | **HR; 95% CI** |  |
| Poor/very poor health (n=5,214) | Face-to-face contact with co-inhabitants | ≥3 people | 4,512/899 | 1.00 | 0.18 | 3,824/248 | 1.00 | 0.03 | 4,449/271 | 1.00 | 0.76 | 4,583/255 | 1.00 | 0.88 |
|  |  | < 3 people | 439/122 | 1.23 (1.01,1.50) * |  | 380/39 | 1.36 (0.95-1.95) |  | 435/27 | 1.00 (0.67-1.48) |  | 449/39 | 1.32 (0.93-1.87) |  |
|  |  | Live alone | 179/57 | 1.47 (1.11-1.95) * |  | 161/27 | 2.56 (1.68-3.90) ** |  | 175/12 | 1.18 (0.65-2.10) |  | 182/15 | 1.21 (0.70-2.11) |  |
|  | Face-to-face contact with non-co-inhabitants | ≥1 time /month | 4,142/838 | 1.00 | 0.98 | 3,538/248 | 1.00 | 0.49 | 4,213/258 | 1.00 | 0.56 | 4,213/241 | 1.00 | 0.64 |
|  |  | < 1 time/month | 774/165 | 1.06 (0.89-1.25) |  | 650/47 | 0.97 (0.70-1.34) |  | 785/44 | 0.95 (0.69-1.31) |  | 785/50 | 1.06 (0.89-1.25) |  |
|  |  | No such contact | 44/19 | 1.54 (0.94-2.54) |  | 38/5 | 1.18 (0.43-3.19) |  | 44/7 | 1.91 (0.84-4.32) |  | 44/6 | 1.54 (0.94-2.54) |  |
|  | Non-face-to-face contact  (by telephone/mail) | ≥1 time /month | 3,587/704 | 1.00 | 0.54 | 3,042/204 | 1.00 | 0.51 | 3,644/213 | 1.00 | 0.09 | 3,644/203 | 1.00 | 0.50 |
|  |  | < 1 time/month | 1,012/212 | 0.93 (0.79-1.09) |  | 864/67 | 0.90 (0.68-1.44) |  | 1,030/59 | 0.88 (0.65-1.18) |  | 1,030/66 | 0.93 (0.79-1.09) |  |
|  |  | No such contact | 364/106 | 1.19 (0.96-1.48) |  | 323/29 | 0.97 (0.65-1.44) |  | 371/37 | 1.47 (1.02-2.12) * |  | 371/28 | 1.19 (0.96-1.48) |  |
|  | Club/organization contact | ≥1 time /month | 2,079/469 | 1.00 | 0.36 | 1,749/135 | 1.00 | 0.53 | 2,116/140 | 1.00 | 0.32 | 2,116/137 | 1.00 | 0.63 |
|  |  | < 1 time/month | 2,878/552 | 1.05 (0.93-1.20) |  | 2,476/165 | 1.09 (0.85-1.38) |  | 2,923/169 | 0.97 (0.77-1.23) |  | 2,923/159 | 1.05 (0.93-1.20) |  |
|  | Composite social isolation score^#^ | 0~7 | 4,960/1,022 | 1.08 (1.02-1.14) * | 0.60 | 4,226/300 | 1.11 (1.00-1.23) | 0.20 | 5,042/309 | 1.07 (0.96-1.19) | 0.66 | 5,042/297 | 1.08 (0.97-1.20) * | 0.88 |
| Good/very good health  (n=24,177) | Face-to-face contact with co-inhabitants | ≥3 people | 22,033/3,396 | 1.00 |  | 20,691/1,097 | 1.00 |  | 21,984/1,202 | 1.00 |  | 22,362/896 | 1.00 |  |
|  |  | < 3 people | 1,372/278 | 1.15 (1.01-1.30) * |  | 1,301/99 | 1.15 (0.93-1.42) |  | 1,375/85 | 1.07 (0.86-1.34) |  | 1,397/79 | 1.20 (0.94-1.51) |  |
|  |  | Live alone | 410/78 | 1.11 (0.87-1.40) |  | 387/30 | 1.19 (0.80-1.76) |  | 412/21 | 0.94 (0.60-1.46) |  | 418/25 | 1.32 (0.87-2.01) |  |
|  | Face-to-face contact with non-co-inhabitants | ≥1 time /month | 21,039/3,235 | 1.00 |  | 19,781/1,062 | 1.00 |  | 20,991/1,146 | 1.00 |  | 21,352/840 | 1.00 |  |
|  |  | < 1 time/month | 2,613/475 | 1.08 (0.98-1.19) |  | 2,444/145 | 0.99 (0.83-1.18) |  | 2,614/153 | 1.00 (0.84-1.18) |  | 2,656/150 | 1.29 (1.08-1.53) * |  |
|  |  | No such contact | 104/30 | 1.68 (1.17-2.41) * |  | 95/14 | 2.47 (1.45-4.19) ** |  | 103/7 | 1.09 (0.52-2.30) |  | 106/6 | 1.28 (0.57-2.87) |  |
|  | Non-face-to-face contact (by telephone/mail) | ≥1 time /month | 18,863/2,821 | 1.00 |  | 17,697/904 | 1.00 |  | 18,816/1,013 | 1.00 |  | 19,145/742 | 1.00 |  |
|  |  | < 1 time/month | 3,657/638 | 1.08 (0.99-1.18) |  | 3,434/208 | 1.06 (0.91-1.24) |  | 3,644/221 | 1.06 (0.92-1.23) |  | 3,707/166 | 1.05 (0.88-1.25) |  |
|  |  | No such contact | 1,230/280 | 1.29 (1.14-1.46) ** |  | 1,186/108 | 1.39 (1.13-1.70) * |  | 1,243/73 | 1.03 (0.81-1.31) |  | 1,257/88 | 1.46 (1.16-1.83) ** |  |
|  | Club/organization contact | ≥1 time /month | 11,126/1,965 | 1.00 |  | 10,431/646 | 1.00 |  | 11,090/686 | 1.00 |  | 11,315/532 | 1.00 |  |
|  |  | < 1 time/month | 12,584/1,769 | 0.98 (0.92-1.05) |  | 11,845/572 | 1.04 (0.92-1.17) |  | 12,573/619 | 0.88 (0.79-0.99) * |  | 12,754/463 | 0.98 (0.86-1.12) |  |
|  | Composite social isolation score^#^ | 0~7 | 23,687/3,726 | 1.08 (1.04-1.12) ** |  | 22,254/1,214 | 1.08 (1.02-1.15) * |  | 23,639/1,304 | 1.02 (0.95-1.08) |  | 24,045/993 | 1.14 (1.07-1.22) ** |  |

* P<0.05; ** P<0.001. HR=hazard ratio; CI=confidence interval. ^#^ Social isolation score was calculated as a continuous variable. † Factors adjusted including sex, age, socioeconomic position, biological factors and behavioral factors.

**Table S9. Associations of face-to-face and non-face-to-face contact with all-cause mortality after mutual adjustment of 3 types of contact.**

| **Social contact isolation types** | **Number/frequency** | **Participants/**  **deaths** | **All-cause**  **mortality** | **Participants/**  **deaths** | **CVD mortality**  **HR; 95% CI** | **Participants/**  **deaths** | **Cancer mortality**  **HR; 95% CI** | **Participants/**  **deaths** | **Other-cause**  **mortality** |
| --- | --- | --- | --- | --- | --- | --- | --- | --- | --- |
|  |  |  | **HR; 95% CI** |  |  |  |  |  | **HR; 95% CI** |
| Face-to-face contact with  co-inhabitants | ≥3 people | 26,920/4,323 | 1.00 | 24,873/1,352 | 1.00 | 26,806/1,485 | 1.00 | 27,322/1,160 | 1.00 |
|  | < 3 people | 1,884/407 | 1.16 (1.04-1.29) * | 1,352/139 | 1.18 (0.99-1.42) | 1,879/115 | 1.05 (0.86-1.28) | 1,919/121 | 1.20 (0.99-1.46) * |
|  | Live alone | 618/134 | 1.10 (0.91-1.34) | 576/54 | 1.41 (1.03-1.92) * | 616/34 | 0.93 (0.64-1.36) | 630/40 | 1.16 (0.81-1.65) |
| Face-to-face contact with  non-co-inhabitants | ≥1 time /month | 25,279/4,030 | 1.00 | 23,428/1,298 | 1.00 | 25,666/1,419 | 1.00 | 25,666/1,073 | 1.00 |
|  | < 1 time/month | 3,434/636 | 1.05 (0.96-1.85) | 3,134/192 | 0.96 (0.82-1.13) | 3,488/201 | 0.97 (0.83-1.14) | 3,488/198 | 1.22 (1.04-1.43) * |
|  | No such contact | 146/46 | 1.35 (1.00-1.85) * | 130/18 | 1.35 (0.82-1.13) | 147/14 | 1.34 (0.75-2.42) | 147/11 | 1.18 (0.64-2.17) |
| Non-face-to-face contact  (by telephone/mail) | ≥1 time /month | 22,536/3,482 | 1.00 | 20,834/1,101 | 1.00 | 22,875/1,238 | 1.00 | 22,875/934 | 1.00 |
|  | < 1 time/month | 4,703/845 | 1.03 (0.95-1.11) | 4,328/273 | 1.02 (0.89-1.18) | 4,771/285 | 1.02 (0.89-1.17) | 4,771/232 | 1.01 (0.87-1.18) |
|  | No such contact | 1,607/381 | 1.23 (1.10-1.38) ** | 1,519/132 | 1.22 (1.01-1.47) * | 1,642/111 | 1.13 (0.92-1.38) | 1,642/115 | 1.30 (1.08-1.62) * |

* P<0.05; ** P<0.001.

HR=hazard ratio; CI=confidence interval.

† Factors adjusted including sex, age, self-rated health, socioeconomic position, biological factors, behavioral factors and face-to-face or non-face-to-face social isolation as appropriate (not included club/organization isolation, which showed no association with mortality).

**Table S10. Associations of social contact isolation with all-cause mortality in original dataset (without excluding 244 death within first 2 years).**

| **Social contact isolation types** | **Number/frequency** | **Participants/**  **deaths** | **Minimal adjusted**  **(HR; 95% CI)** | **Fully adjusted**  **(HR; 95% CI)** | **P for trend**  **(fully adjusted model)** |
| --- | --- | --- | --- | --- | --- |
| Face-to-face contact with  co-inhabitants | ≥3 people | 27,497/4,595 | 1.00 | 1.00 | <0.001 |
|  | < 3 people | 1,918/436 | 1.23 (1.11-1.35) * | 1.18(1.07-1.31) * |  |
|  | Live alone | 640/146 | 1.25 (1.05-1.48) * | 1.21 (1.01-1.44) * |  |
| Face-to-face contact with  non-co-inhabitants | ≥1 time /month | 26,046/4,377 | 1.00 | 1.00 | <0.001 |
|  | < 1 time/month | 3,527/688 | 1.09 (1.00-1.18) * | 1.07 (0.98-1.16) |  |
|  | No such contact | 158/54 | 1.76 (1.34-2.31) ** | 1.63 (1.23-2.15) ** |  |
| Non-face-to-face contact  (by telephone/mail) | ≥1 time /month | 23,212/3,784 | 1.00 | 1.00 | <0.001 |
|  | < 1 time/month | 4,844/914 | 1.08 (1.00-1.16) * | 1.05 (0.97-1.13) |  |
|  | No such contact | 1,671/419 | 1.34 (1.21-1.49) ** | 1.36 (1.22-1.51) ** |  |
| Club/organization contact | ≥1 time /month | 13,643/2,599 | 1.00 | 1.00 | 0.480 |
|  | < 1 time/month | 16,033/2,510 | 1.03 (0.97-1.09) | 0.94 (0.89-1.00) |  |
| Composite social isolation score^#^ | 0~7 | 29,715/5,113 | 1.10 (1.07-1.13) ** | 1.08 (1.05-1.11) ** |  |

* P<0.05; ** P<0.001.

^#^ Social contact isolation score was analyzed as a continuous variable.

HR=hazard ratio; CI=confidence interval.

Minimal adjusted model: adjusted for sex, age and self-rated health.

Fully adjusted model: adjusted for sex, age, self-rated health, socioeconomic position, biological factors (body mass index, systolic blood pressure, diastolic blood pressure and fasting glucose) and behavioral factors (smoking status, alcohol use and physical activity)


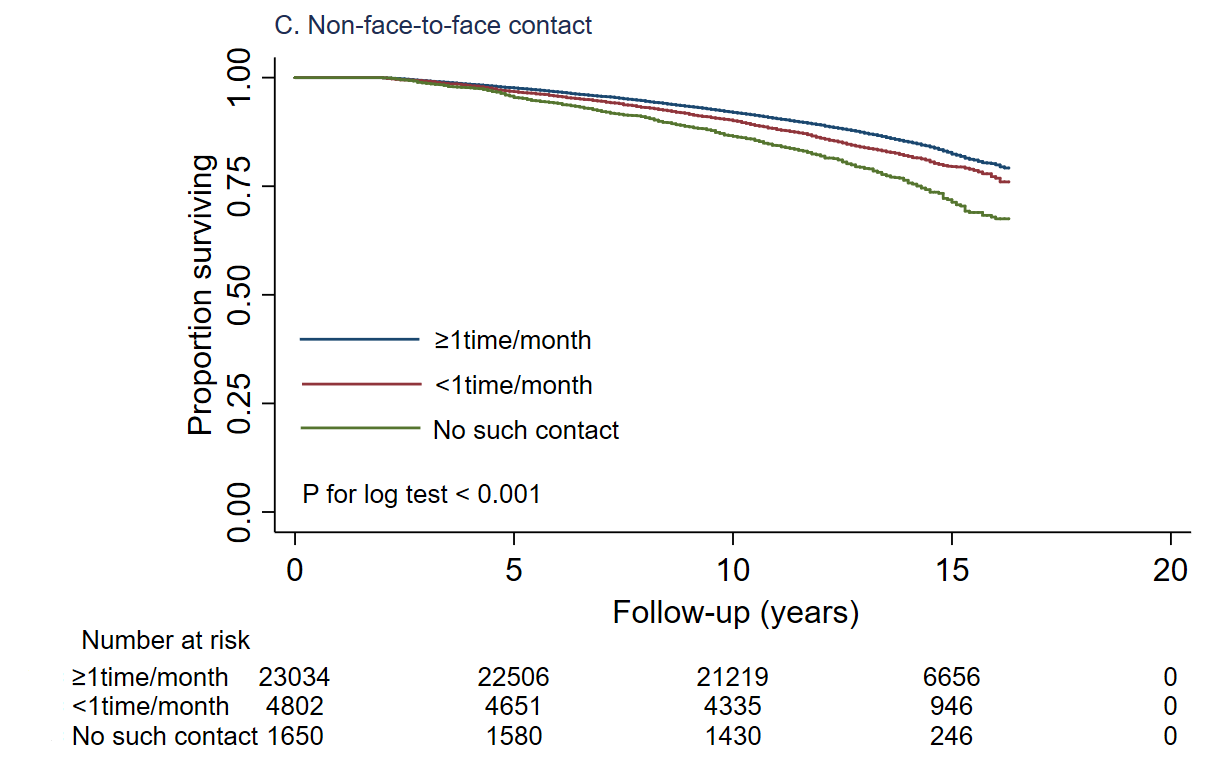

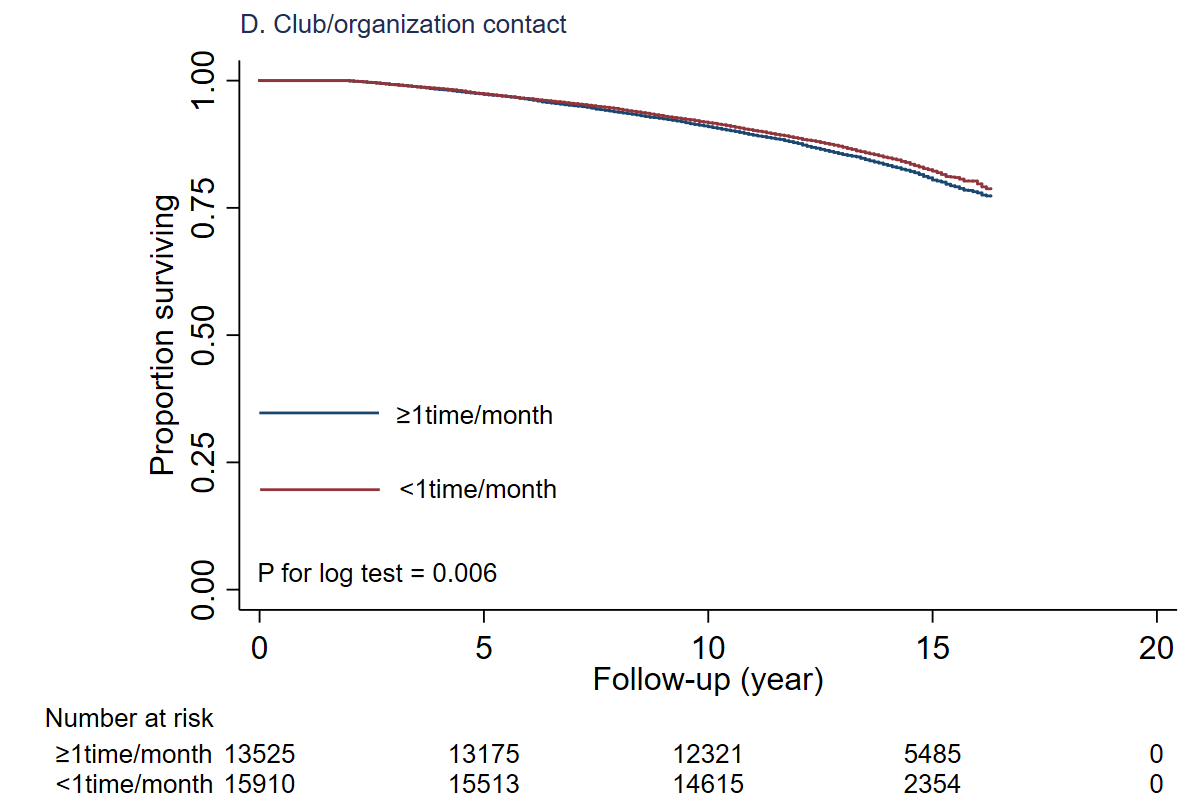

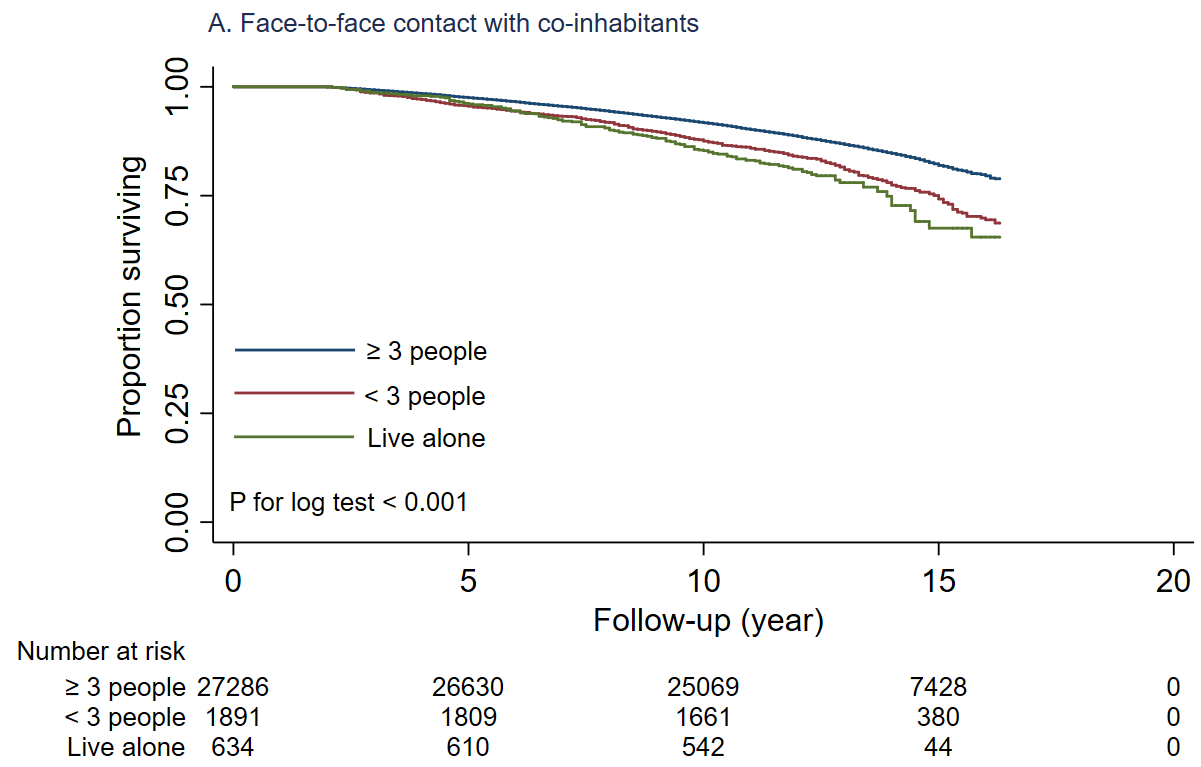

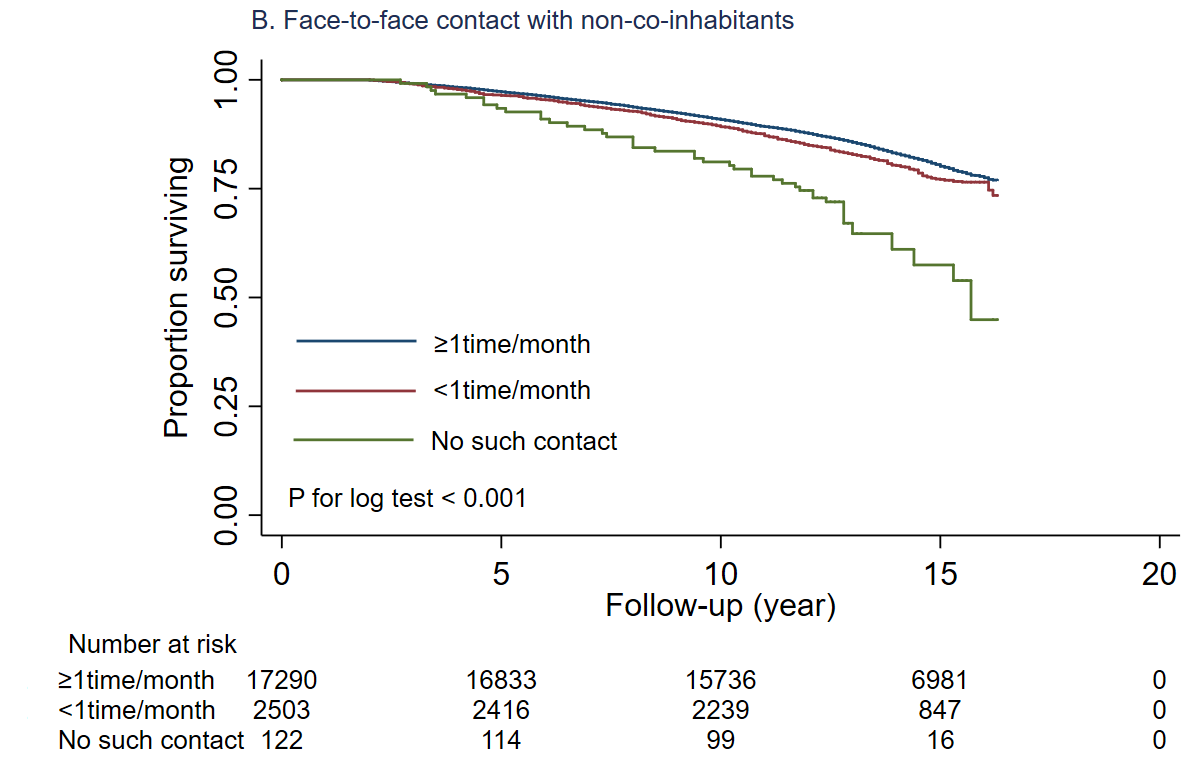


**Figure S1.** **Kaplan–Meier survival curve by four types of social isolation assessed at baseline examination (2003-2008) for all-cause mortality in 30,430 participants.**

A: Kaplan–Meier survival curve by face-to-face contact with co-inhabitants for all-cause mortality. B: Kaplan–Meier survival curve by face-to-face contact with non-co-inhabitants for all-cause mortality.

C: Kaplan–Meier survival curve by non-face-to-face contact (by telephone/mail) for all-cause mortality. D: Kaplan–Meier survival curve by club/organization contact for all-cause mortality.


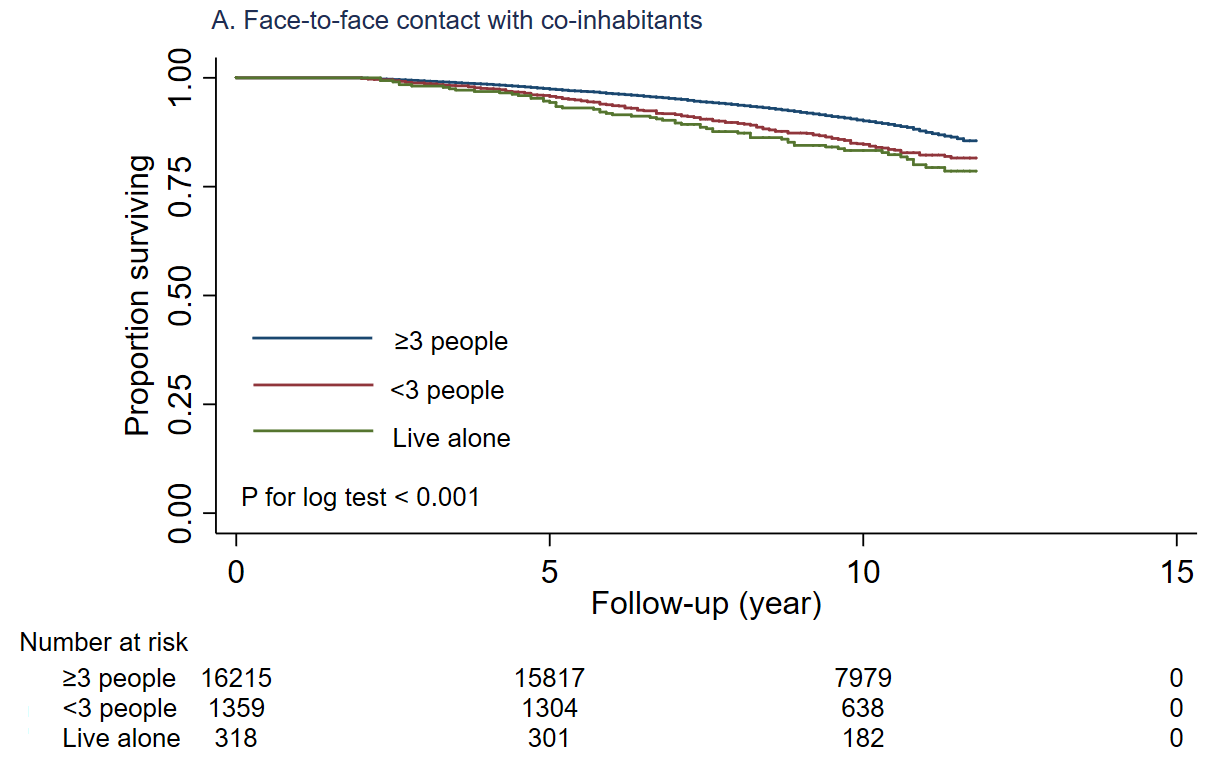

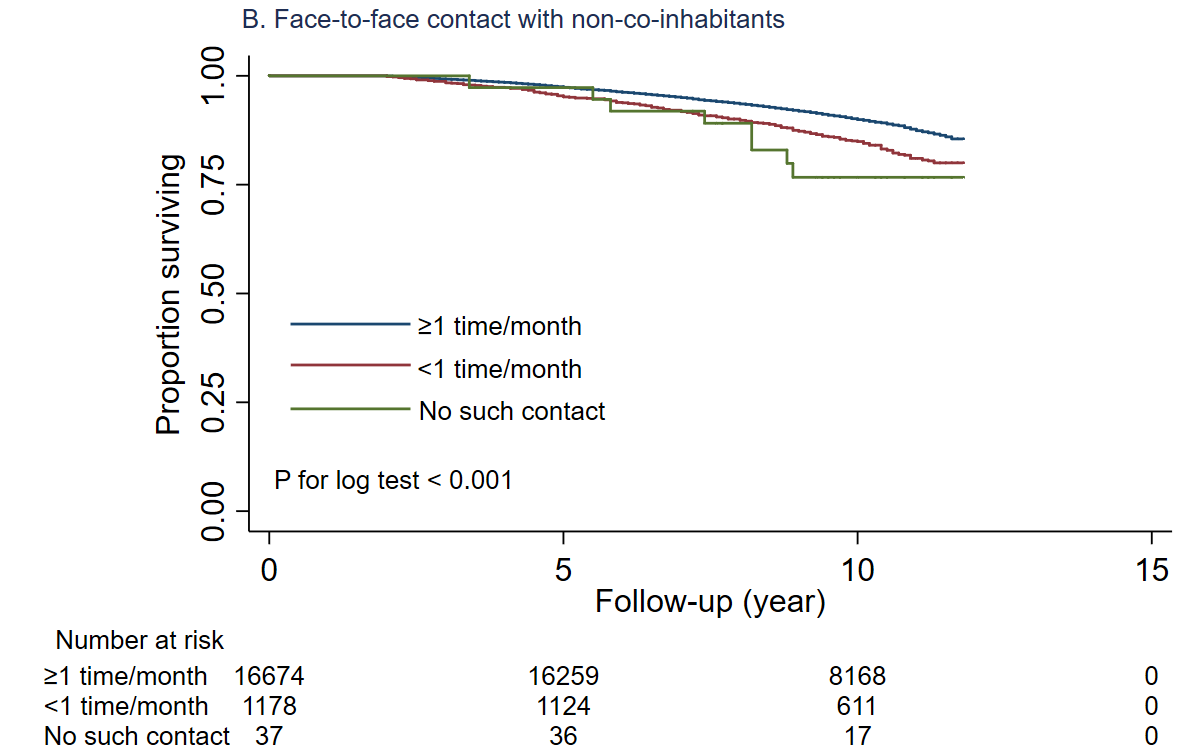

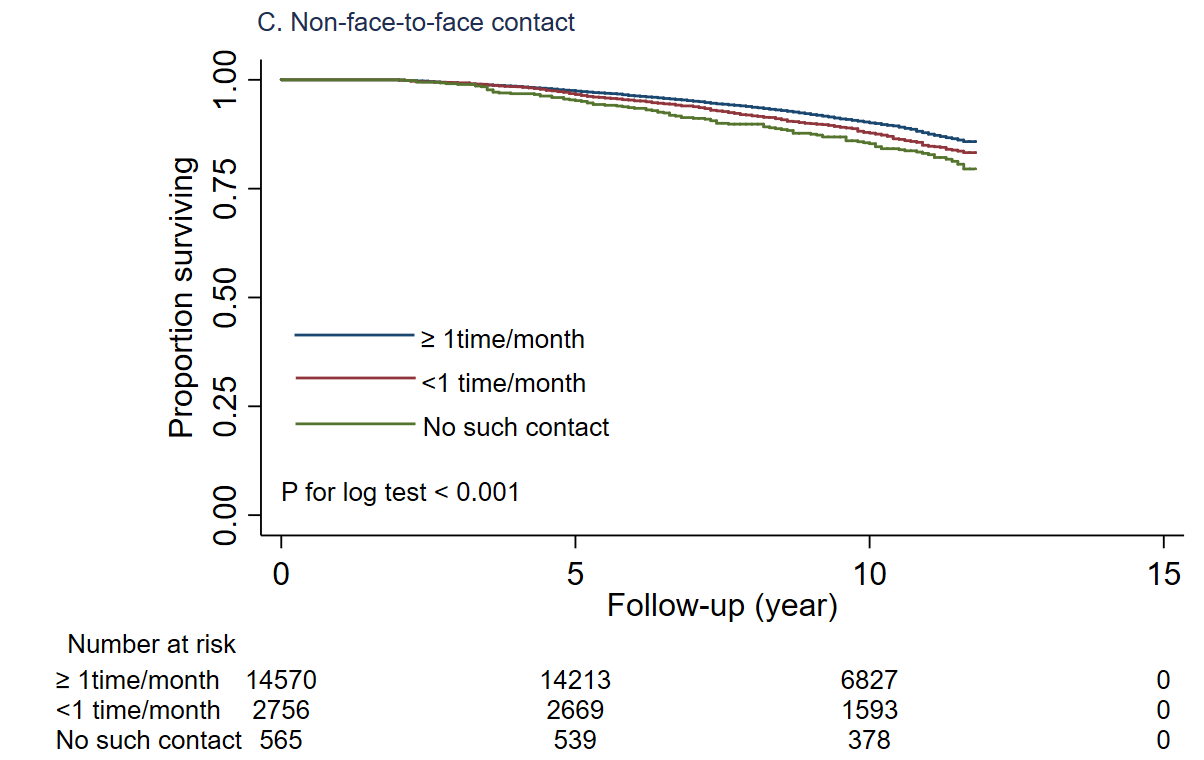

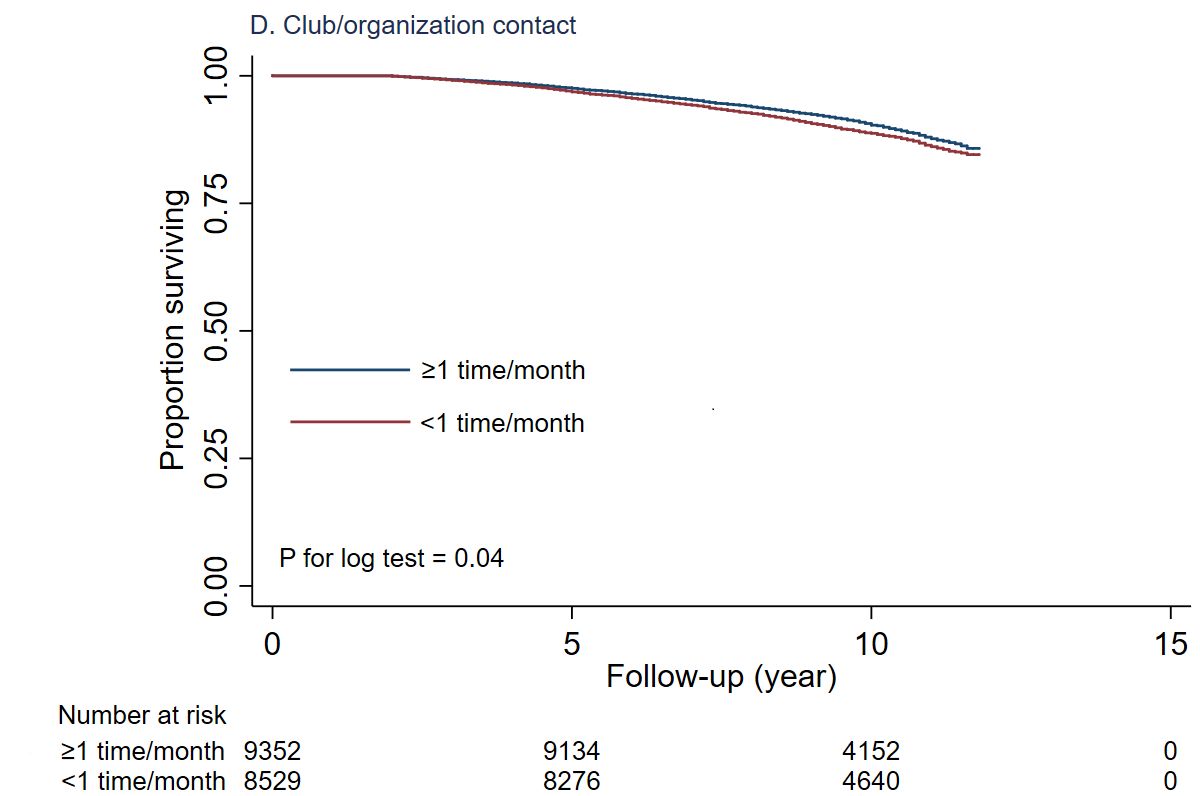


**Figure S2. Kaplan–Meier survival curve by four types of social isolation assessed at follow-up examination (2008-2012) for all-cause mortality in 18,104 participants.**

A: Kaplan–Meier survival curve by face-to-face contact with co-inhabitants for all-cause mortality. B: Kaplan–Meier survival curve by face-to-face contact with non-co-inhabitants for all-cause mortality. C: Kaplan–Meier survival curve by non-face-to-face contact (by telephone/mail) for all-cause mortality. D: Kaplan–Meier survival curve by club/organization contact for all-cause mortality.
